# *De novo* recovery of Ghana virus, an African bat Henipavirus, reveals differential tropism and attenuated pathogenicity compared to Nipah virus

**DOI:** 10.1016/j.celrep.2026.117074

**Published:** 2026-03-12

**Authors:** Griffin D. Haas, Olivier Escaffre, Rebecca A. Reis, Terry L. Juelich, Jennifer K. Smith, Lihong Zhang, Birte K. Kalveram, Axel A. Guzmán-Solís, Dariia Vyshenska, William Klain, Alexander L. Greninger, Alexander N. Freiberg, Benhur Lee

**Affiliations:** 1 Department of Microbiology, Icahn School of Medicine at Mount Sinai, New York, NY, USA; 2 Department of Pathology, University of Texas Medical Branch, Galveston, TX, USA; 3 Department of Microbiology and Immunology, University of Texas Medical Branch, Galveston, TX, USA; 4 Department of Laboratory Medicine and Pathology, University of Washington, Seattle, WA, USA; 5 Lead contact

## Abstract

Henipaviruses (HNVs) like Nipah virus (NiV) and Hendra virus (HeV) represent severe zoonotic threats. Ghana virus (GhV), identified in 2012, is the only African bat henipavirus with a near-complete genome assembly. However, without isolates in culture, GhV biology, pathogenicity, and zoonotic potential remain poorly understood. Using reverse genetics, we recovered a full-length infectious clone of GhV at BSL-4 following rational reconstruction of its incomplete 3′ leader and modification of a non-canonical transcriptional initiation site. GhV demonstrated restricted receptor tropism (ephrin-B2 but not ephrin-B3) and distinct innate immune antagonism. Replication was attenuated in primary human cells but was enhanced in bat cells. In Syrian golden hamsters, GhV infection caused no disease or mortality. Furthermore, a chimeric NiV encoding the GhV receptor-binding protein was completely attenuated *in vivo*, implicating ephrin-B3 receptor usage as a critical determinant of HNV pathogenesis. These findings elucidate GhV zoonotic potential and inform strategies for virus surveillance and control.

## In brief

African henipaviruses have not been isolated in culture; using reverse genetics, Haas et al. recover an infectious clone of Ghana virus and characterize its restricted receptor usage, replication across cell types, antiviral susceptibility, and attenuation *in vivo*. They further demonstrate that loss of ephrin-B3 receptor usage attenuates Nipah virus pathogenicity.

## Graphical Abstract

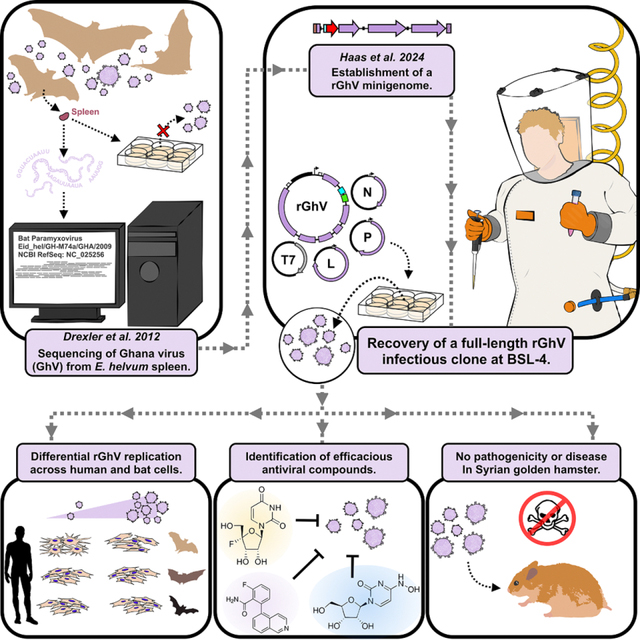

## INTRODUCTION

Bats are reservoirs of paramyxoviruses, including highly pathogenic henipaviruses (HNVs) such as Nipah virus (NiV) and Hendra virus (HeV).^1–4^ Spillover events of NiV and HeV have been geographically restricted to Southeast Asia and Australia, corresponding to the range of their reservoir, Pteropid (*Pteropus* genus) bats.^5,6^ However, wildlife surveillance indicates that HNVs are globally distributed, with significant evidence of HNV circulation in African fruit bats, particularly *Eidolon helvum* (*E*. *helvum*).^7–13^ Given that an estimated 128,000 *E*. *helvum* bats are captured annually as bushmeat in Ghana alone, there are concerns that African bat HNVs could spill over into humans.^14^ In a cohort from Cameroon, seropositivity to HNVs was exclusively observed in individuals involved in butchering bats, suggesting that such spillover events are already occurring.^11^

In 2012, a novel HNV genome was identified from the spleen of an *E*. *helvum* bat, leading to the discovery of Ghana virus (GhV, strain M74a).^1^ To date, GhV remains the only available genome of an African bat HNV, and it has yet to be isolated in culture. Consistent with *E*. *helvum* serving as a reservoir species for GhV, a recent study reported a high seroprevalence of neutralizing antibodies against GhV pseudoviruses in *E*. *helvum* bats (66.7%; 36/54).^13^ Although there is an abundance of serological and metagenomic evidence for African bat henipaviruses,^7–13^ no additional full-length GhV genomes or closely related GhV-like sequences have been reported since its discovery, highlighting a persistent gap between evidence of HNV circulation and recovery of complete viral genomes. The dearth of isolates for GhV has hindered experimental interrogation of its biology, pathogenicity, and potential risk to human health.

A combination of receptor usage and potent antagonism of the host innate immune response is thought to underlie the severe disease caused by prototypical HNVs.^15,16^ NiV and HeV use highly conserved ephrin-B2 (EFNB2) and ephrin-B3 (EFNB3) receptors to enter cells.^17–19^ EFNB2 is more ubiquitously expressed across neurons, endothelial cells, and the smooth muscle of arterial vessels, while EFNB3 expression is largely restricted to the central nervous system; this tissue distribution corresponds to the tropism and neurovirulence of NiV and HeV.^20,21^ By contrast, experimental binding and structural studies suggest that GhV only utilizes EFNB2.^22^ However, the *in vivo* consequences of this more restricted receptor usage have not been explored. Furthermore, NiV and HeV encode multiple virulence factors that suppress host antiviral responses during infection.^23,24^ HNVs generate antagonists of innate immune signaling by co-transcriptional editing of the phosphoprotein gene.^23,24^ These antagonists, “V” and “W,” are generated when the vRdRp inserts one or two non-templated guanosine nucleosides, respectively, into nascent P-gene transcripts at a characteristic editing site. In NiV, these proteins serve as key antagonists: NiV-P binds STAT1/2 to inhibit interferon (IFN)-mediated signaling, V suppresses cytosolic antiviral sensing pathways such as MDA5, and W traffics to the nucleus where it blocks the induction of IFN-stimulated genes.^25–28^ It remains unknown whether the corresponding V and W orthologs in GhV are expressed or are functionally sufficient to antagonize the human innate immune response.

In this study, we used reverse genetics to recover a full-length, infectious clone of GhV *de novo* at BSL-4. Recovery of GhV required restoration of its missing genomic promoter and modification of a non-canonical gene start (GS) motif upstream of the M gene. GhV replication was attenuated relative to NiV in primary human and porcine cells, but certain bat cell lines supported more robust GhV replication. Experimental infection of Syrian golden hamsters with GhV resulted in no disease or mortality. Furthermore, we demonstrate that EFNB3 receptor usage is a major determinant of HNV pathogenesis by rescuing a recombinant NiV (rNiV) chimera encoding the GhV receptor binding protein (RBP), which was completely attenuated *in vivo*. This work provides experimental characterization of full-length, replication-competent GhV and establishes a framework for future studies of its biology, host range, pathogenicity, and zoonotic potential.

## RESULTS

### The GS encoded upstream of the matrix gene in GhV is non-canonical and does not drive protein expression in a GhV minigenome system

We previously demonstrated that the available genome sequence of GhV M74a is incomplete, lacking 28 nucleotides at the extreme 3′ terminal end spanning its genomic promoter; however, substitution of this missing sequence with the homologous promoter element from HeV was sufficient to facilitate recognition by the GhV RNA-dependent RNA polymerase (vRdRp) in minigenome assays.^29^ Building upon this approach, we constructed and rescued a full-length recombinant Ghana virus (rGhV) reverse genetics system encoding a *Gaussia* luciferase (GLuc) and eGFP reporter cassette between the N and P genes (Figure S1A). Initial attempts to rescue this virus produced GFP-positive foci by 11 days post-transfection (DPT); however, by 20 DPT, expansion of these foci was minimal, and spread appeared to be predominantly driven by cell-to-cell contact, producing few if any distal infection events (Figure S1B).

**Figure 1.**
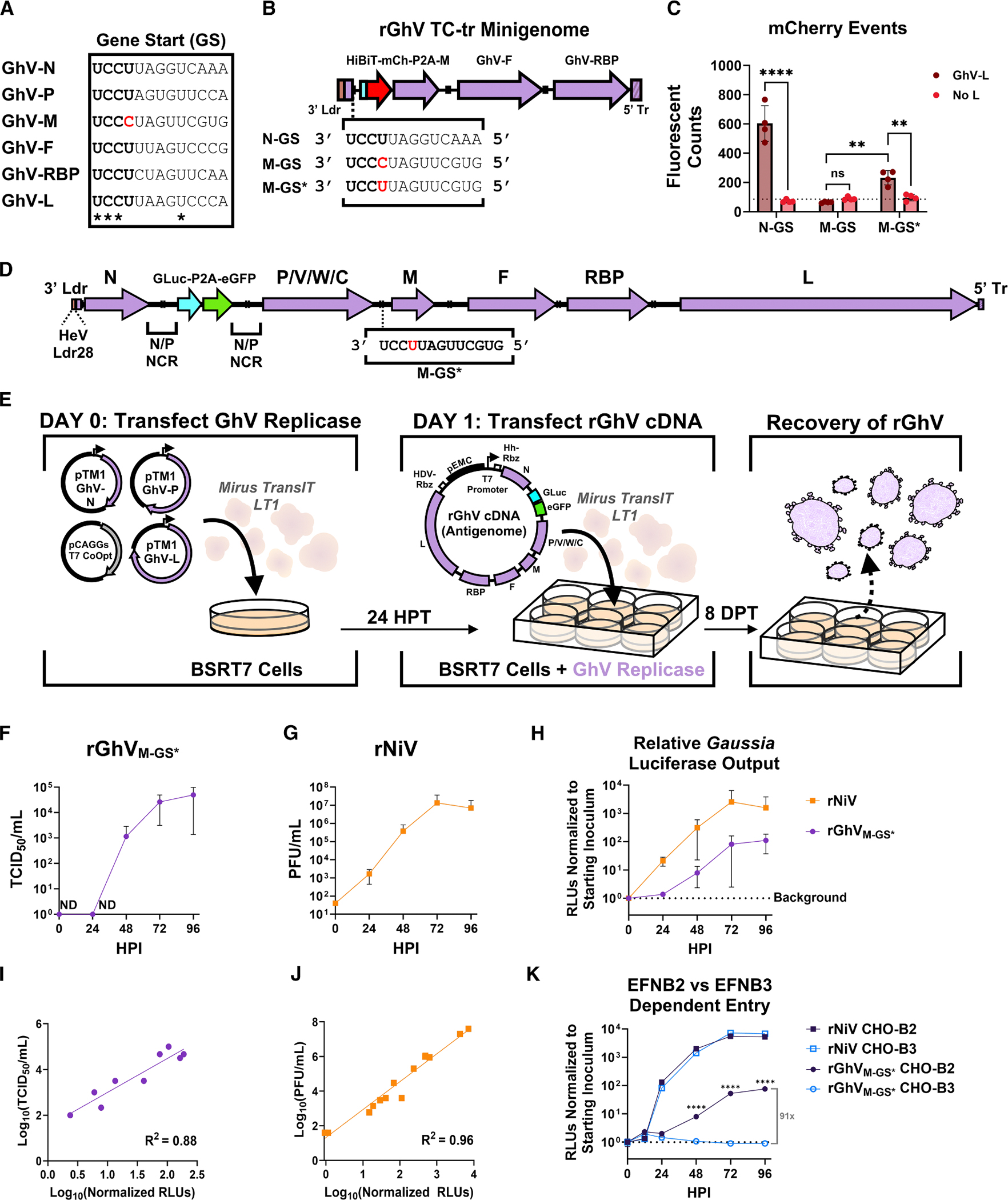
Restoring a canonical GS in GhV strain M74a enables rescue and amplification of rGhV *de novo* (A) Alignment of gene start (GS) sequences encoded upstream of each gene in GhV M74a. The “UCCU” consensus motif is shown in bold, and absolutely conserved bases are denoted with an asterisk below the alignment. The non-canonical base in the GhV-M GS is highlighted in red. (B) Design of rGhV minigenomes in which the reporter gene is encoded under the N-GS, M-GS, or a modified M-GS (M-GS*), respectively; the GS sequences controlling reporter expression are shown next to each label, and the non-canonical base that is changed in M-GS* is highlighted in red. (C) Quantification of mCherry-positive events from each minigenome in the presence or absence of GhV-L. See also Figures S3B and S3C. (D) Schematic of the rGhV_M-GS*_ genome. (E) Schematic detailing the rescue of rGhV_M-GS*_. (F) Growth of rGhV_M-GS*_ in CHO-B2 cells, with viral titers determined by TCID_50_ assay. ND indicates no detectable virus. (G) Growth of rNiV in CHO-B2 cells measured by plaque assay. (H) Corresponding *Gaussia* luciferase activity from supernatants in (F) and (G), with RLUs normalized to the signal from the 0 HPI timepoint for each respective virus. Correlation between log_10_(titer) and log_10_(normalized RLUs) for rGhV_M-GS*_ (purple) (I) and rNiV (orange) (J) was used to determine the reliability of *Gaussia* luciferase as a surrogate for virus replication. (K) Relative replication of rNiV (squares) and rGhV_M-GS*_ (circles) in CHO-B2 cells (dark blue) and CHO-B3 cells (light blue) measured by *Gaussia* luciferase assays. RLUs were normalized to the starting inoculum (0 HPI) for each respective virus. Statistical significance was determined by two-way ANOVA with Šídák’s multiple comparisons test in GraphPad Prism to compare relative infection in CHO-B2 and CHO-B3 at each time point. Non-significant datapoints are unlabeled (*p* > 0.05); *p ≤ 0.05, **p ≤ 0.01, ***p ≤ 0.001, and ****p ≤ 0.0001. All experiments were conducted in biological triplicate, and error bars represent standard deviation.

Comprehensive *in silico* analyses indicated that the coding sequences (CDS) of each gene in GhV were consistent with the expected HNV gene architecture (Figure S2), so we next examined the reference genome for potential assembly variations in intergenic regions that might affect viral replication efficiency. To achieve expression, each viral gene must be flanked by an upstream GS sequence and a downstream gene end (GE) sequence.^30–32^ Sequence alignment of bat-borne HNV GS sequences demonstrated an absolutely conserved 3′-UCCU-5′ motif localized immediately downstream of each intergenic region (IGR), while GE sequences consisted of a highly conserved 3′-AAUNNNUUUU-5′ motif. Based on these consensus motifs, we were able to identify putative GS and GE sequences flanking each gene (Figure S3A). Curiously, the GS upstream of the matrix gene (GhV-M) began with the motif 3′-UCCC-5′ and was the sole GS observed to break the otherwise conserved 3′-UCCU-5′ consensus (Figures 1A and S3A).

To investigate whether this non-canonical motif impacted GS activity, we utilized our previously described rGhV minigenome.^29^ We generated three constructs in which the reporter gene was encoded under the control of either the GhV-N GS, the reported native GhV-M GS, or a modified GhV-M GS (denoted as M-GS*) in which we made a single nucleotide change to restore the 3′-UCCU-5′ consensus motif (Figure 1B). The GhV-N GS was sufficient to drive significant expression of the HiBiT-mCherry reporter gene by the vRdRp, yielding several hundred mCherry-positive cells over the no-L control (Figure 1C). However, when the M-GS was employed, we observed no mCherry-positive cells over the no-L control. Notably, the single-nucleotide change in the minigenome encoding M-GS* was sufficient to restore partial activity, resulting in significant, albeit modest, expression of the reporter gene over the no-L control (Figures 1C, S3B, and S3C).

### A rGhV encoding an M-GS bearing the consensus motif (M-GS*) can be rescued and amplified in culture, albeit with delayed kinetics relative to NiV

The minigenome assay suggested that the non-canonical M-GS likely impairs M protein expression and may contribute to the inability of full-length rGhV to amplify in culture. We encoded a single nucleotide change in the M-GS in our full-length rGhV infectious clone, such that the GhV-M gene was instead under control of the demonstrated functional M-GS* (Figure 1D). BSR-T7/5 cells were co-transfected with plasmids encoding the viral replicase and, 24 h later, with plasmid encoding the rGhV antigenome (rGhV_M-GS*_) (Figure 1E). By 8 DPT, GFP-positive foci were observed throughout the well, with obvious instances of particle and cell-to-cell spread (Figure S3D). rGhV_M-GS*_ stocks were amplified, validated by RNA sequencing, and titrated by TCID_50_ on highly permissive Chinese hamster ovary (CHO) cells that over-express the HNV entry receptor, EFNB2 (these cells are referred to as CHO-B2). RNA sequencing confirmed that the HeV genomic promoter, used in lieu of the unmapped GhV genomic promoter, remained unchanged during virus amplification.

To assess replication kinetics, CHO-B2 cells were infected with either rGhV_M-GS*_ or rNiV (Malaysia strain), with both viruses encoding a GLuc-P2A-eGFP reporter cassette. Supernatants from infected cells were collected at the indicated time points for virus titration and quantification of secreted *Gaussia* luciferase. rGhV_M-GS*_ showed exponential growth and achieved an endpoint titer of 5 × 10^4^ TCID_50_/mL by 96 h post-infection (HPI) with an eclipse phase of >24 h (Figure 1F). By contrast, rNiV infection of CHO-B2 cells yielded measurable progeny virus as early as 24 HPI (Figure 1G). Delayed replication kinetics of rGhV_M-GS*_ but not rNiV were consistent with prior minigenome observations (Figure S4). *Gaussia* luciferase activity reflected the titration data at each time point (Figure 1H), demonstrating a strong positive correlation between relative light units (RLUs) and virus titer for both rGhV_M-GS*_ (Figure 1I) and rNiV (Figure 1J). Thus, *Gaussia* luciferase serves as a reliable surrogate for measuring viral replication.

We previously demonstrated that GhV utilizes EFNB2, but not EFNB3, for entry into cells.^22^ To validate GhV receptor usage, CHO cells expressing either EFNB2 (CHO-B2) or EFNB3 (CHO-B3) were infected with rGhV_M-GS*_ or rNiV, and supernatants from infected cells were collected at the indicated time points for *Gaussia* luciferase detection. While rNiV could replicate equally well in both CHO-B2 and CHO-B3 cells, rGhV_M-GS*_ could only replicate in CHO-B2 cells (91-fold increase) (Figure 1K). In both cell types, rNiV resulted in widespread cytopathic effect (CPE) and formed large syncytia, consistent with its highly fusogenic F-RBP complex; by contrast, rGhV_M-GS*_ infection yielded predominantly single-cell infection events, with only occasional, modest syncytia (Figures S5A–S5D). These observations are consistent with reports demonstrating that GhV glycoproteins are less fusogenic than NiV glycoproteins.^33–37^

### Co-transcriptional editing of the GhV-P gene yields products that antagonize human innate immune signaling in a manner distinct from NiV

To characterize the transcriptional profile of GhV, we employed nanopore long-read direct RNA sequencing on total RNA from rGhV_M-GS*_ infected CHO-B2 cells. Mapping of mRNA reads to the rGhV_M-GS*_ antigenome revealed that during infection, the vRdRp produces a characteristic 3′–5′ transcriptional gradient, where the number of polyadenylated transcripts (mRNAs) encoded N > P > M > F > RBP > L (Figure S6A). This sequencing further confirmed our predicted gene boundaries in Figures 1 and S3A.

**Figure 2.**
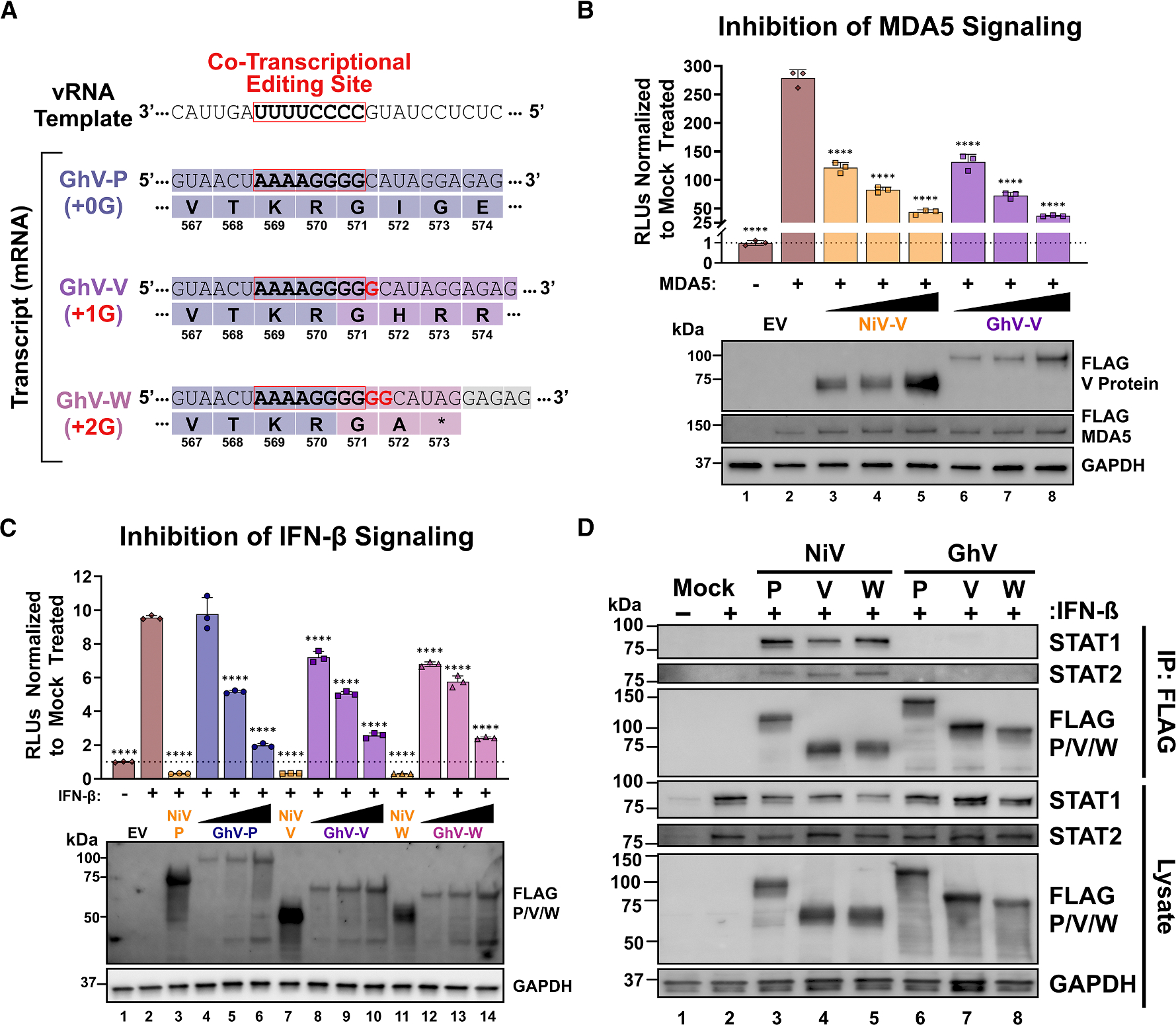
Co-transcriptional editing of the GhV-P gene yields products that antagonize human innate immune signaling in a manner distinct from NiV (A) Schematic of the co-transcriptional editing site encoded in the GhV-P gene. Insertion of a single non-templated guanosine nucleoside at the editing site yields GhV-V, and the insertion of two non-templated guanosines yields GhV-W. Non-templated nucleosides are depicted in red. (B) Inhibition of MDA5-driven signaling by NiV-V and GhV-V measured by dual-luciferase reporter assay in HEK-293T cells. (C) Inhibition of IFN-β–driven signaling by NiV-P, -V, and -W and GhV-P, -V, and -W in HEK-293T cells. For both (B) and (C), RLUs were normalized to the untreated EV control. (D) Co-immunoprecipitation of endogenous STAT1 and STAT2 by either FLAG-tagged NiV-P, -V, and -W or GhV-P, -V, and -W. Western blots in (B) and (C) confirm the expression of the indicated proteins in each condition, with GAPDH serving as a loading control. Statistical significance in (B) and (C) was assessed by one-way ANOVA with Dunnett’s multiple comparisons test, comparing each condition to the respective EV control. Non-significant data points (*p* > 0.05) are not labeled; *p ≤ 0.05, **p ≤ 0.01, ***p ≤ 0.001, and ****p ≤ 0.0001. All experiments were conducted in biological triplicate, with error bars representing standard deviation.

The GhV-P gene encodes a canonical editing site and retains the coding potential to make V and W proteins (Figure 2A). Analysis of reads mapped to the editing site revealed that co-transcriptional editing does occur during rGhV_M-GS*_ infection, with 79% of transcripts encoding P, 13% encoding V, and 8% encoding W (Figures S6B and S6C). Although derived from a single replicate, the proportion of P-edited transcripts (V + W = 21%) appeared to be lower than the >50% of P-edited transcripts produced by NiV and HeV.^23,38,39^ This is likely an underestimate of the true editing frequency, as homopolymeric sequences of >4 bases are known to be consistently underestimated on the Oxford Nanopore direct RNA-seq platform.^40^ To determine if the P-editing products of GhV can antagonize human innate immune signaling, we cloned FLAG-tagged P, V, and W proteins from NiV and GhV into an expression vector for dual luciferase reporter assays. These assays are widely used to quantify the activation or inhibition of interferon-signaling pathways by measuring firefly luciferase expression under an IFN-responsive promoter normalized to a constitutively expressed Renilla luciferase control.^25,26,28^ We observe that both GhV-V and NiV-V potently inhibited IFN-β promoter activation of innate immune signaling via MDA5, corroborating experimental evidence and *in silico* predictions of MDA5 binding (Figures 2B and S6D). We further observed that the GhV-P, -V, and -W proteins drove a dose-dependent inhibition of transcription from an interferon-stimulated response element (ISRE) promoter during IFN-β treatment (Figure 2C). We pulled down FLAG-tagged P, V, and W proteins and blotted for human STAT1 and STAT2 to determine if the mechanism of IFN-β signaling antagonism is conserved between NiV and GhV. While we detected co-immunoprecipitation of human STAT1 and STAT2 by NiV-P, -V, and -W, there was no detectable pull-down of these factors by GhV-P, -V, and -W (Figure 2D). The described STAT1-binding motif in NiV-P is poorly conserved in GhV-P, supporting the conclusion that GhV antagonism occurs in a manner independent of STAT1 binding (Figure S6E).

### Primary human and porcine cells support limited replication of rGhV_M-GS*_

**Figure 3.**
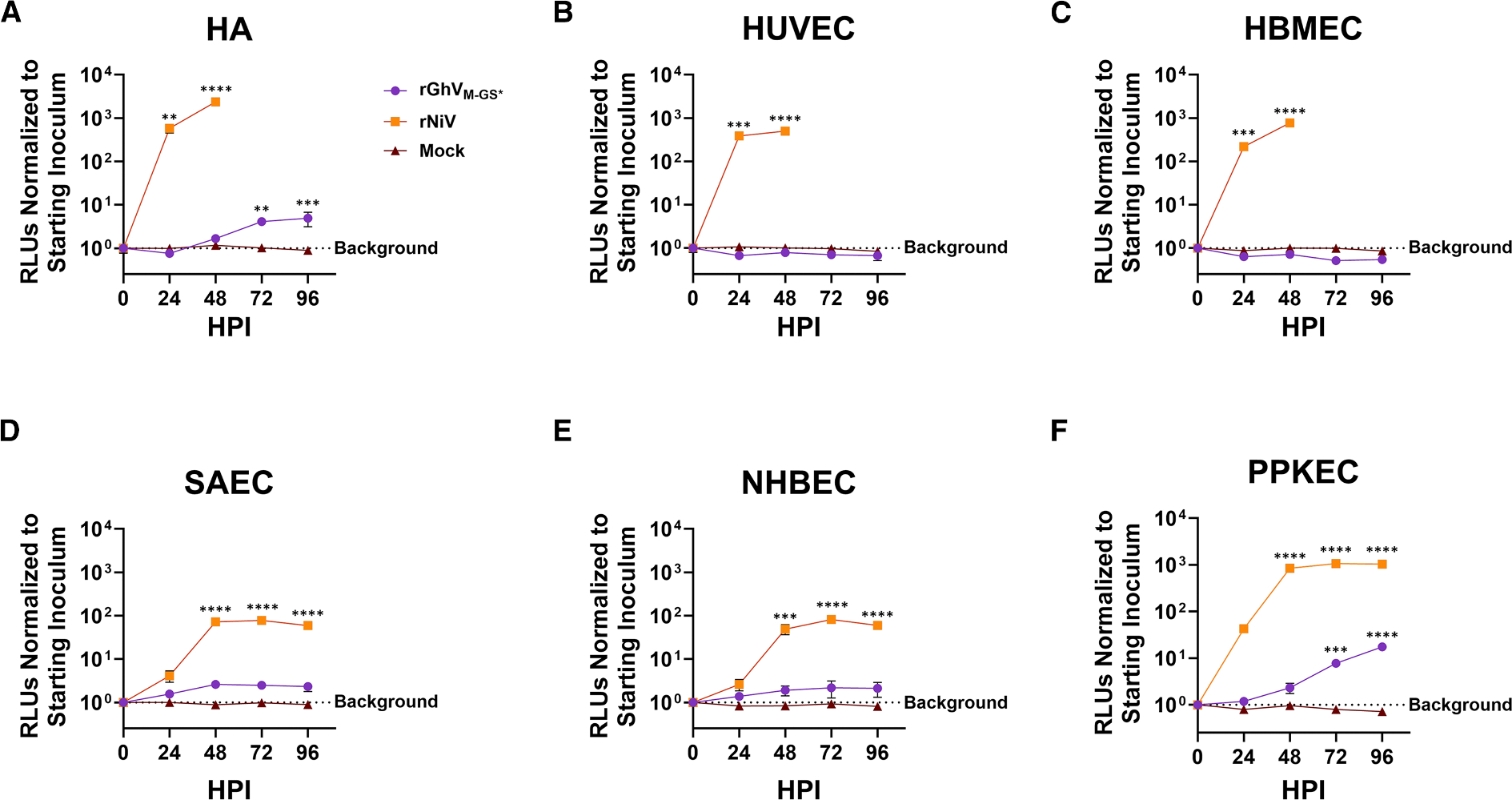
Primary human and porcine cells support limited rGhV_M-GS*_ replication Replication kinetics of rGhV_M-GS*_ (purple), rNiV (orange), and mock-infected (brown) (A) primary human astrocytes (HAs), (B) human umbilical vein endothelial cells (HUVECs), (C) human brain microvascular endothelial cells (HBMECs), (D) small airway epithelial cells (SAECs); (E) normal human bronchial epithelial cells (NHBECs), and (F) primary porcine kidney epithelial cells (PPKECs), measured by *Gaussia* luciferase activity. RLUs in the supernatants were measured and normalized to the 0 HPI time point for each condition. Statistical significance was determined by one-way ANOVA in GraphPad Prism with Dunnett’s multiple comparisons test to compare normalized RLUs at each time point to baseline RLUs at 0 HPI for each virus. Non-significant data points (*p* > 0.05) are not labeled; *p ≤ 0.05, **p ≤ 0.01, ***p ≤ 0.001, and ****p ≤ 0.0001. Virus infections were conducted in at least biological triplicate, and mock infections were conducted in at least biological duplicate, with error bars representing standard deviation.

To better understand the zoonotic potential of GhV, we assessed its ability to replicate in a panel of primary human and porcine cells relevant to NiV pathology. rNiV replicated more robustly than rGhV_M-GS*_ in primary human astrocytes (HAs), achieving >2,000-fold increase in normalized RLUs (nRLU) compared to less than 10-fold increase in nRLUs for rGhV_M-GS*_ by 96 HPI (Figure 3A). Nonetheless, rGhV_M-GS*_ clearly infected HAs, producing small syncytia (Figure S7A). By contrast, while rNiV replicated efficiently in both primary human umbilical vein endothelial cells (HUVECs, 499-fold increase in RLUs) and primary human brain microvascular endothelial cells (HBMECs, 775-fold increase in RLUs), rGhV_M-GS*_ showed no detectable signs of infection and was apparently restricted in these cells (Figures 3B and 3C). Similarly, albeit to a lesser extent, normal human bronchial epithelial cells (NHBECs, Figure 3D) and small airway epithelial cells (SAEC, Figure 3E) supported robust rNiV replication (81-fold and 78-fold increases in RLUs, respectively), but limited rGhV_M-GS*_ replication. Primary porcine kidney epithelial cells (PPKECs) supported both rNiV replication (1,066-fold increase in RLUs) and moderate rGhV_M-GS*_ replication (17-fold increase in RLUs) (Figure 3F). Matched fluorescence microscopy data for Figure 3 are presented in Figure S7.

### A panel of bat cell lines supports differential rGhV and rNiV infection

**Figure 4.**
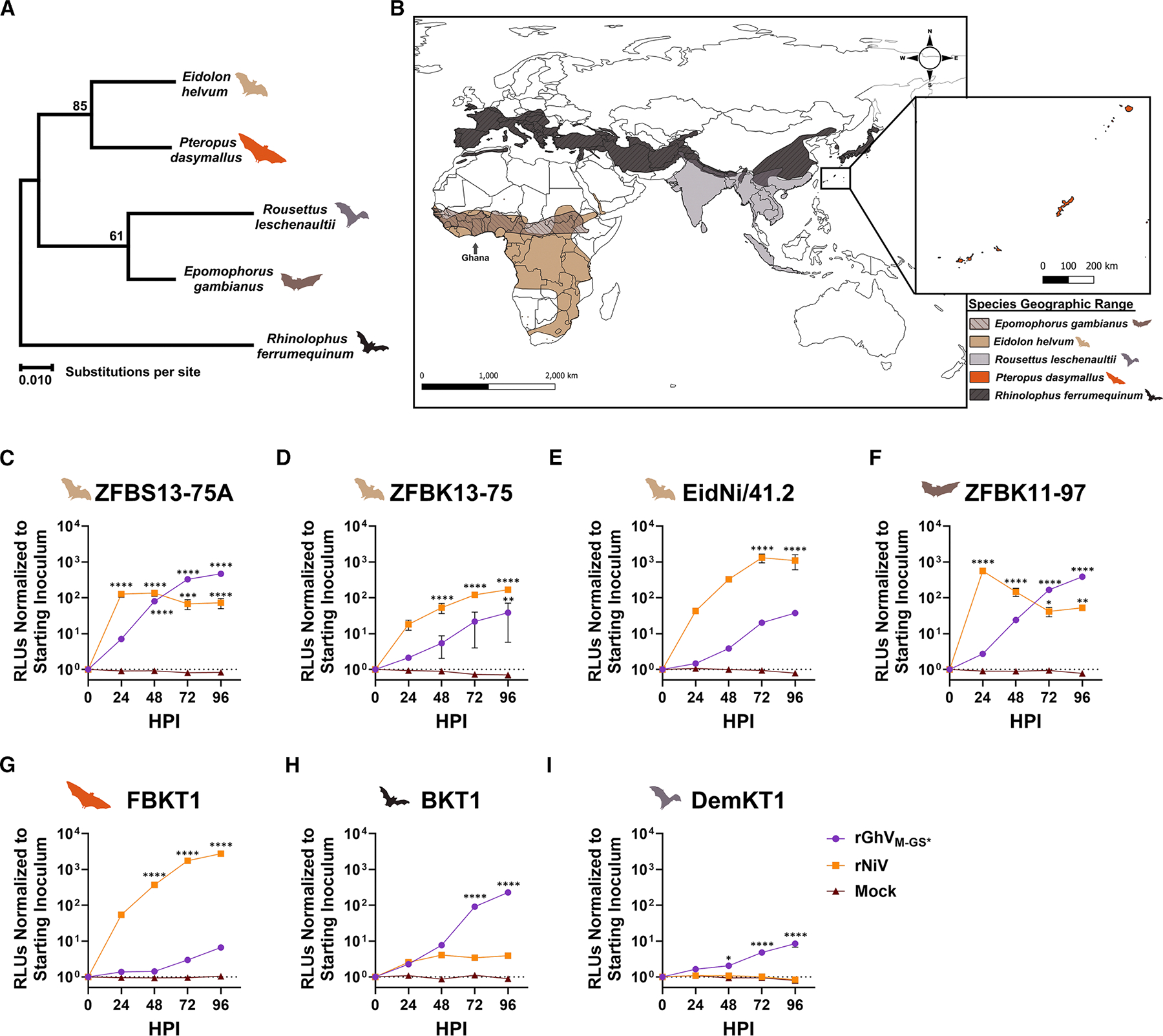
A panel of bat cells supports differential replication of rGhV_M-GS*_ and rNiV (A) Phylogenetic tree based on the cytochrome *b* gene from representative bat species in this study. (B) Geographical range of respective bat species. Colored/shaded areas indicate species distribution ranges based on International Union for Conservation of Nature (IUCN) data (https://www.iucnredlist.org), with *Eidolon helvum* depicted in tan; *Epomophorus gambianus* depicted in striped brown, *Pteropus dasymallus* depicted in orange (its range is shown in the inset); *Rousettus leschenaultii* depicted in light gray; and *Rhinolophus ferrumequinum* depicted with striped dark gray. The location of Ghana is marked with an arrow. (C– I) Replication kinetics of rGhV_M-GS*_ (purple), rNiV (orange), or mock-infected (brown) bat cell lines derived from (C–E) *Eidolon helvum*, (F) *Epomophorus gambianus*, (G) *Pteropus dasymallus*, (H) *Rhinolophus ferrumequinum*, and (I) *Rousettus leschenaultii*, measured by *Gaussia luciferase* assay. RLUs were normalized to the 0 HPI time point for each virus. Statistical significance was determined by two-way ANOVA in GraphPad Prism with Šídák’s multiple comparisons test to compare normalized RLUs at each time point with the corresponding RLUs at 0 HPI for each virus. Non-significant datapoints are unlabeled (*p* > 0.05); *p ≤ 0.05, **p ≤ 0.01, ***p ≤ 0.001, and ****p ≤ 0.0001. All experiments were conducted in biological triplicate, and error bars represent standard deviation.

We next evaluated rGhV_M-GS*_ and rNiV replication across a panel of bat-derived cell lines. These cells, representing five distinct bat species, were infected with either rNiV or rGhV_M-GS*_ and monitored for signs of infection as in Figure 3. The bat species included in this panel, their phylogeny, and geographical distribution are depicted in Figures 4A and 4B.

*E*. *helvum* cells supported appreciable rNiV replication, with peak increases in nRLUs of 126-fold, 167-fold, and 1303-fold in spleen-derived ZFBS13–75A (Figure 4C), kidney-derived ZFBK13–75 (Figure 4D), and kidney-derived EidNi/41.2 (Figure 4E), respectively. ZFBS13–75A cells supported robust rGhV_M-GS*_ replication, yielding a 468-fold peak increase in nRLUs that was almost 4× higher than rNiV (Figure 4C). rGhV_M-GS*_ replication was more moderate in ZFBK13–75 cells and in EidNi/41.2 cells, yielding 38-fold and 37-fold increases in nRLUs, respectively (Figures 4D and 4E).

*E*. *gambianus* kidney-derived ZFBK11–97 cells supported robust replication of both rNiV and rGhV_M-GS*_, with 558-fold and 386-fold peak increases in nRLUs, respectively (Figure 4F). *P*. *dasymallus* kidney-derived FBKT1 cells supported robust rNiV replication (2,746-fold increase) with only minimal rGhV_M-GS*_ replication (7-fold) (Figure 4G). Interestingly, two other bat kidney cell lines supported better replication of rGhV_M-GS*_ than rNiV. *Rhinolophus ferrumequinum* (greater horseshoe bat) BKT1 cells supported more robust rGhV_M-GS*_ replication than rNiV, with a peak 227-fold vs. 4-fold increase in nRLUs, respectively (Figure 4H). *Rousettus leschenaultii* DemKT1 cells supported low-level replication of rGhV_M-GS*_ (an 8-fold increase in RLUs) but entirely restricted rNiV replication (Figure 4I).

To determine if receptor expression accounts for the differential susceptibility of bat cell lines to NiV and GhV, we quantified the relative abundance of *EFNB2* transcripts from each cell line by quantitative reverse-transcription PCR (RT-qPCR) and plotted their relative expression levels against the peak normalized luciferase (nLuc) values for rGhV_M-GS*_ and rNiV, respectively (Figures S8A and S8B). While there appears to be a moderate correlation between relative *EFNB2* expression and peak nRLUs for rGhV_M-GS*_, this was not true for rNiV (Figures S8A and S8B). Matched fluorescence microscopy corroborates the infection data in Figure 4 (Figures S8C–S8I). Alignment of the EFNB2 and EFNB3 G-H loop regions from all bat species used in this study revealed complete conservation of the residues required for RBP engagement (Figures S8J–S8K). *EFNB3* expression was uniformly low across these cells except in DemKT1 cells and is unlikely to play a role in the differential susceptibility patterns seen, especially since GhV does not use EFNB3 for entry^22^ (Figure S8L). Collectively, these data strongly suggest post-entry compatibilities drive the differential infection phenotypes observed between rNiV and rGhV_M-GS*_ in cells such as BKT1 and DemKT1.

### Experimental infection of Syrian golden hamsters with rGhV_M-GS*_ does not cause disease or mortality

**Figure 5.**
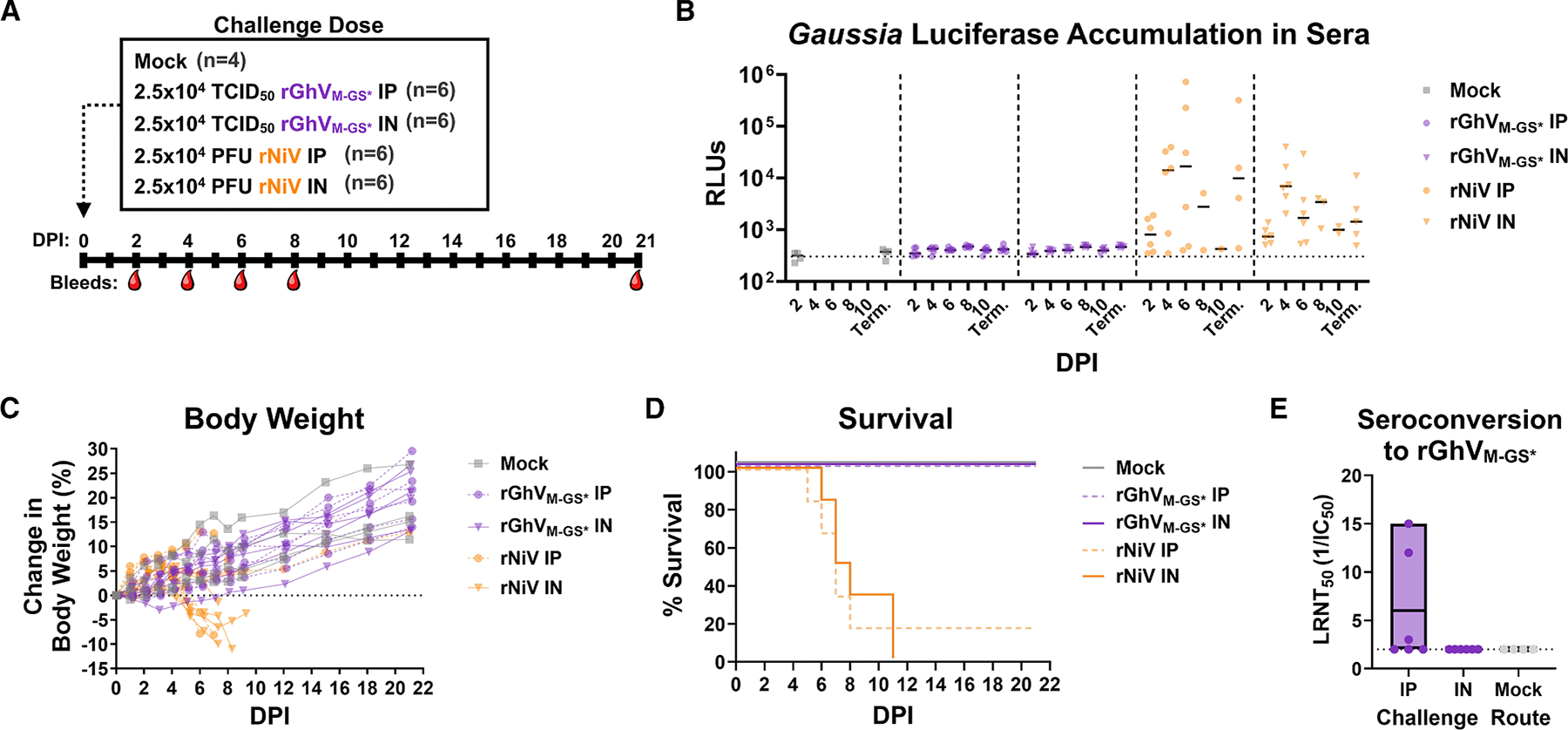
rGhV_M-GS*_ challenge in Syrian Golden Hamster animal model (A) Schematic of the challenge design. Groups of Syrian golden hamsters were inoculated either intraperitoneally (IP) or intranasally (IN) with 2.5 × 10^4^ TCID_50_ of rGhV_M-GS*_ (*n* = 6 per group) or 2.5 × 10^4^ PFU of rNiV (*n* = 6 per group). Mock-infected (*n* = 4) animals served as controls. Retro-orbital (RO) bleeds were collected at 2, 4, 6, and 8 DPI, with terminal bleeds collected at the time of euthanasia or at 21 DPI. (B) Accumulation of *Gaussia* luciferase in serum as a surrogate for measuring virus replication in animals. Sera from RO and terminal bleeds were used for GLuc assays. (C) Percent change in body weight over time. (D) Survival curves of hamsters challenged with rGhV_M-GS*_ or rNiV compared to mock. (E) Quantification of luciferase neutralizing antibody titers against rGhV_M-GS*_ (LRNT_50_) in terminal sera from rGhV_M-GS*_ or mock-challenged animals. For all graphs, each datapoint represents an individual animal. Mock is depicted in gray (rectangles), rGhV_M-GS*_ is depicted in purple, and rNiV is depicted in orange. IP groups are depicted as circles, and IN groups are depicted as triangles.

The Syrian golden hamster is a robust animal model for HNV disease and pathogenesis.^41,42,43^ To evaluate the pathogenicity of GhV, we experimentally infected Syrian golden hamsters (*n* = 6 per group) with either 2.5 × 10^4^ TCID_50_ of rGhV_M-GS*_ or 2.5 × 10^4^ plaque-forming unit (PFU) of rNiV (back-titrated at 1.38 × 10^4^ PFU). Both viruses were administered either intraperitoneally (IP) or intranasally (IN). A mock-infected control group (*n* = 4) was maintained in parallel. Animals were observed daily for clinical signs of illness, and retro-orbital (RO) bleeds were collected at the indicated time points (Figure 5A). *Gaussia* luciferase from viral replication can be measured in serum from RO and terminal bleeds.^11,44,45^ While hamsters infected with rNiV demonstrated increases in RLUs relative to the mock control as early as 2 DPI, hamsters infected with rGhV_M-GS*_ exhibited no appreciable rise in RLUs at any time point (Figure 5B). Consistent with this observation, hamsters infected with rGhV_M-GS*_ experienced no significant weight loss (Figure 5C) nor mortality (Figure 5D). By contrast, animals infected with rNiV experienced significant weight loss between 4 and 8 DPI (Figure 5C), with 6/6 animals succumbing to infection in the IN challenge group, and 5/6 succumbing to infection in the IP challenge group (Figure 5D). To confirm that hamsters had been successfully infected with rGhV_M-GS*_, terminal sera collected at 21 DPI from survivors were tested in neutralization assays against rGhV_M-GS*_. Although none of the IN infected animals seroconverted, 3/6 animals infected IP developed detectable neutralizing antibody titers above the limit of detection (Figure 5E), and neutralizing activity was observed only in seropositive animals.

### A chimeric, EFNB3-blind rNiV is completely attenuated in Syrian golden hamsters

**Figure 6.**
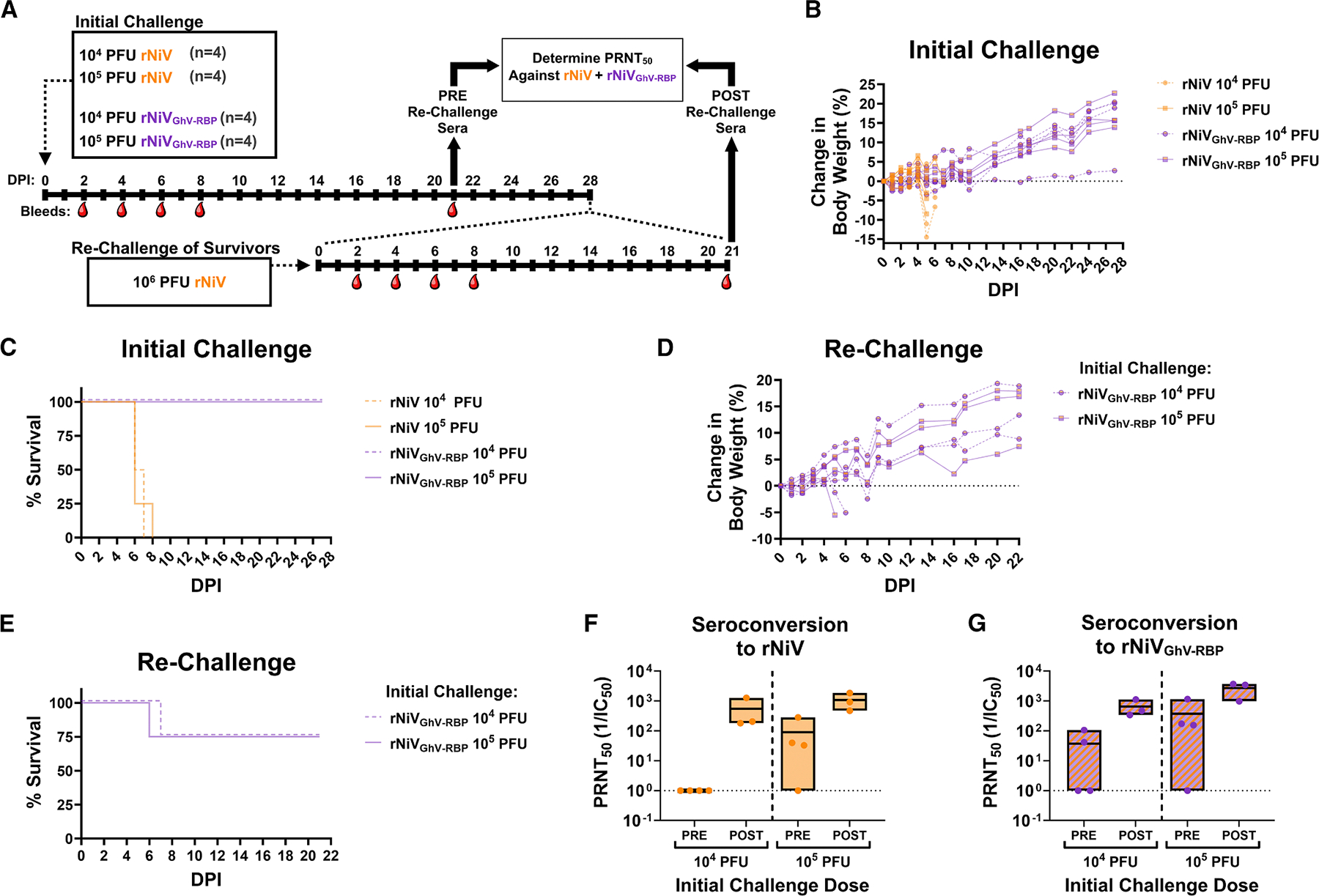
Syrian golden hamster challenge with rNiV and rNiV_GhV-RBP_ (A) Schematic of the two-phase animal challenge. Syrian golden hamsters (*n* = 4 per group) were initially challenged with either 10^4^ PFU or 10^5^ PFU of rNiV or chimeric rNiV encoding the GhV-RBP (rNiV_GhV-RBP_). RO bleeds were collected at 2, 4, 6, 8, and 21 DPI. At 28 DPI, survivors were re-challenged with 10^6^ PFU of rNiV, with RO bleeds collected at 2, 4, 6, 8, and 21 days post re-challenge. Sera collected at 21 days post initial challenge (PRE re-challenge) and at 21 days post re-challenge (POST re-challenge) were used to determine seroconversion by PRNT_50_. (B) Percent change in body weight over time following the initial challenge with rNiV (orange) or rNiV_GhV-RBP_ (half-orange, half-purple). Each data point represents an individual animal; circles indicate a dose of 10^4^ PFU, and squares depict a dose of 10^5^ PFU. (C) Survival curves from the initial challenge. (D) Percent change in body weight over time following the re-challenge of survivors with 10^6^ PFU of rNiV. Datapoint depictions reflect the initial challenge. (E) Survival curves from the re-challenge. (F and G) Neutralizing antibody titers (PRNT_50_) against (F) rNiV and (G) rNiV_GhV-RBP_ were determined from sera collected PRE and POST re-challenge, respectively. Floating bar plots indicate the minimum and maximum values, with the horizontal line representing the mean.

To evaluate the role of differential receptor usage (EFNB2 vs. EFNB3) in HNV pathogenicity, we constructed a chimeric rNiV clone in which the NiV-RBP was replaced with the CDS of the GhV-RBP (Figure S9A). We anticipated that this virus would be viable, as previous studies indicated that the GhV-RBP could complement NiV-F in a fusion assay.^46,47^ The chimeric virus, rNiV_GhV-RBP_, was rescued and amplified in culture and demonstrated kinetics comparable to the isogenic wild-type (WT) rNiV (Figure S9B). Furthermore, the chimeric virus was unable to infect CHO-B3 cells (Figure S9C). To determine the effect of GhV-RBP on pathogenicity, Syrian golden hamsters (*n* = 4 per group) were challenged intraperitoneally with 1 × 10^4^ or 1 × 10^5^ PFU of rNiV_GhV-RBP_ (back-titrated at 2.23 × 10^4^ and 1.8 × 10^5^ PFU) or WT rNiV (back-titrated at 1.1 × 10^4^ and 8.67 × 10^4^ PFU). Animals were monitored for health and weight loss, with RO bleeds collected at the indicated timepoints. At 21 DPI, all survivors were bled, and at 28 DPI, surviving animals were re-challenged with 1 × 10^6^ PFU of WT rNiV to assess whether prior infection conferred protective immunity (Figure 6A).

Animals challenged with WT rNiV exhibited significant weight loss (Figure 6B), with all animals succumbing to infection regardless of the challenge dose (Figure 6C). Conversely, animals challenged with rNiV_GhV-RBP_ developed no clinical signs of disease, with no weight loss (Figure 6B) and no mortality (Figure 6C). Re-challenge of the rNiV_GhV-RBP_ infected survivors with WT rNiV (dosage back-titrated to be 1.13 × 10^5^ PFU) resulted in weight loss and mortality for 1/4 animals from each challenge group; however, the remaining 3/4 animals exhibited no appreciable weight loss nor clinical signs of disease following re-challenge (Figures 6D and 6E). Serum collected from survivors at 21 DPI (PRE rechallenge) and at 21 days post rechallenge with rNiV (POST rechallenge) was analyzed for neutralizing titers. Following the initial rNiV_GhV-RBP_ infection, serum collected 21 DPI (pre-rechallenge) had neutralizing activity against WT rNiV. After rechallenge with WT rNiV, neutralizing antibody titers against rNiV increased markedly in all animals, regardless of the initial inoculation dose (Figure 6F). Likewise, pre-rechallenge sera also neutralized the rNiV_GhV-RBP_ chimera, and rechallenge with WT rNiV virus further boosted neutralizing titers (Figure 6G).

Hamsters challenged with 10^5^ PFU of rNiV_GhV-RBP_ developed neutralizing antibodies against NiV and HeV pseudoviruses by 21 DPI (Figure S10A), and sera collected from survivors after rechallenge had a robust, broadly neutralizing response against NiV, HeV, and GhV pseudoviruses with no detectable neutralization of the VSV-G pseudovirus control (Figure S10B). Flow cytometric analysis of surface antibody binding confirmed reactivity to NiV-F, HeV-F, and GhV-RBP in post-rechallenge sera (Figure S10C). Antibodies targeting NiV-F were cross-reactive only to HeV-F, and anti-GhV-RBP antibodies were not cross-reactive to NiV/HeV-RBP (Figure S10C). These findings indicate that independent polyclonal antibody responses directed against both F and RBP were generated over the course of infection and rechallenge. Together, these independent responses broadened the spectrum of neutralization to include prototypical henipaviruses (NiV and HeV) as well as the GhV-RBP-bearing chimera.

### A panel of antivirals demonstrates efficacy against rGhV_M-GS*_

**Figure 7.**
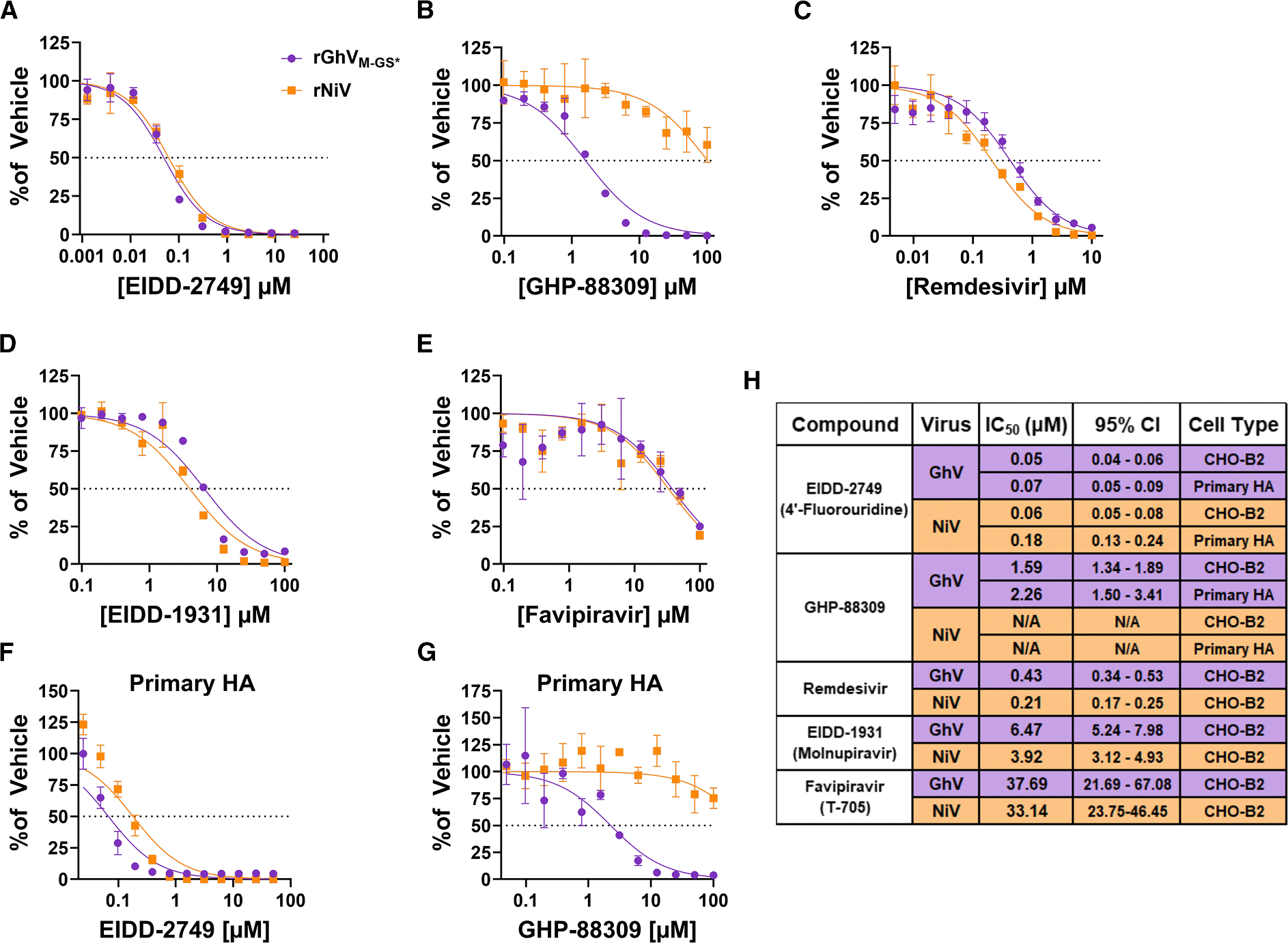
Antiviral activity of small molecule inhibitors against rGhV_M-GS*_ and rNiV (A–E) Dose-response curves are shown for various small-molecule inhibitors against rGhV_M-GS*_ (purple) and rNiV (orange). CHO-B2 cells were infected at an MOI of 0.05 and treated with (A) EIDD-2749, (B) GHP-88309, (C) remdesivir, (D) EIDD-1931 (molnupiravir active form), or (E) favipiravir (T-705). (F and G) Additional dose-response curves were measured in primary human astrocytes for (F) EIDD-2749 and (G) GHP-88309 under the same MOI and assay conditions. (H) Summary of half-maximal inhibitory concentrations (IC_50_) derived from the dose-response curves in (A–G). Each row indicates the respective compound, virus, calculated IC_50_, corresponding 95% confidence interval, and the cell type in which the assay was performed. IC_50_ values were determined in GraphPad Prism by nonlinear regression of (inhibitor) vs. normalized response. All experiments were conducted in biological triplicate. Error bars depict standard deviation.

Because African bat HNVs have not been isolated in culture, their susceptibility to broad-spectrum antiviral compounds is largely unknown. We evaluated the efficacy of several broad-spectrum antivirals using inhibition assays against rGhV_M-GS*_ and rNiV in CHO-B2 cells. EIDD-2749 (4′-fluorouridine), previously shown to inhibit henipaviruses and other paramyxoviruses,^48^ potently inhibited both rNiV and rGhV_M-GS*_ replication, yielding sub-micromolar half-maximal inhibitory concentrations (IC_50_) values (0.05 and 0.06 μM, respectively) (Figure 7A). Recapitulating our prior findings with rGhV TC-tr minigenomes,^29^ GHP-88309, an allosteric inhibitor of paramyxovirus vRdRp,^49^ was effective against rGhV_M-GS*_ (IC_50_ of 1.59 μM) but showed little if any inhibition of rNiV (Figure 7B). Remdesivir, a broad-spectrum nucleoside analog with reported activity against NiV,^50^ inhibited both rNiV and rGhV_M-GS*_ with IC_50_ of 0.21 and 0.43 μM, respectively (Figure 7C). EIDD-1931 (the active form of molnupiravir) inhibited both rNiV (IC_50_ = 3.92 μM) and rGhV_M-GS*_ (IC50 = 6.47 μM) (Figure 7D). Favipiravir (T-705), previously demonstrated to protect hamsters from NiV infection,^51^ demonstrated efficacy against both rNiV (IC50 = 33.14 μM) and rGhV_M-GS*_ (IC50 = 37.69 μM) (Figure 7E). In primary HAs, EIDD-2749 inhibited both rNiV and rGhV_M-GS*_ with similar potency (Figure 7F), whereas GHP-88309 selectively inhibited rGhV_M-GS*_ but showed little to no activity against rNiV (Figure 7G), consistent with the CHO-B2 cell data. A summary of all IC_50_ values derived in this study is presented in Figure 7H.

## DISCUSSION

Difficulties in isolating viruses from environmental samples have hindered our understanding of African bat HNV biology, pathogenesis, and potential zoonotic risk. In this study, we recovered an infectious clone of GhV M74a using reverse genetics, building on our prior work identifying a functional genomic promoter for this virus.^29^ Initial rescue attempts based on the reference sequence yielded a virus with limited amplification, and we demonstrate that this sequence encodes a non-canonical, non-functional GS sequence upstream of the matrix gene. However, restoring the GS consensus motif (M-GS*) enabled efficient rescue and amplification of rGhV. This virus, rGhV_M-GS*_, could be rescued, amplified in culture, and was capable of both cell-to-cell and particle spread.

The V and W proteins of NiV have been experimentally demonstrated as critical drivers of pathogenicity in animal models, and their counterparts in GhV may likewise influence the course of disease during infection.^52,53^ This study confirms that GhV encodes and expresses functional antagonists of human antiviral signaling. While P, V, and W from NiV have a well-established mechanism of antagonizing IFN-β signaling through binding of STAT1 and STAT2,^25,26,54^ our co-immunoprecipitation results suggest that antagonism of IFN-β signaling by GhV antagonists occurs independently of STAT1/STAT2 binding. Further work is needed to better elucidate the unique mechanisms underlying GhV antagonism of IFN-β signaling.

Replication of rGhV_M-GS*_ is characteristically delayed and attenuated relative to rNiV. In primary HAs, rGhV_M-GS*_ replication was detectable yet modest, while infection was completely restricted in HUVECs and HBMECs. Because NiV infection of the brain microvasculature is thought to facilitate entry into the central nervous system, our findings suggest that GhV may be unable to effectively cross the blood-brain barrier. PPKECs, however, supported moderate replication of rGhV_M-GS*_. Because domestic pigs served as amplifying hosts during the initial outbreak of NiV in Malaysia and Singapore,^55,56^ our findings raise concerns that pigs may provide a similar niche for GhV and other bat-borne HNVs. Consistent with this possibility, agricultural serosurveys in Africa have identified seropositivity toward NiV in pigs, suggesting that HNV spillover into livestock may already be occurring.^57–59^

Our infection studies suggest that GhV replicates more efficiently in certain bat-derived cell lines than in non-chiropteran cells. Notably, *E*. *helvum* spleen-derived ZFBS13–75A cells supported robust rGhV_M-GS*_ replication without the kinetics delay observed in non-chiropteran cells (Figure 4C). Given that GhV was identified in *E*. *helvum* spleen, this cellular context may represent a particularly permissive environment for GhV replication.^1^ Similarly, *E*. *gambianus* ZFBK11–97 cells exhibited robust replication and pronounced GhV CPE (Figures 4F and S8F); this is consistent with previous studies demonstrating that GhV fusogenicity may be restricted to specific chiropteran cell lines.^34,35,37^ Although *E*. *gambianus* is sympatric with *E*. *helvum*, serological data show low reactivity to NiV antigen in *E*. *gambianus* (1%; 1/89), in sharp contrast to high seroprevalence in *E*. *helvum* (39%; 23/59),^7^ suggesting species-specific differences in exposure or susceptibility.

Other bat species investigated in this study do not share an overlapping geographic range with *E*. *helvum*. Nonetheless, our findings offer insights into the relative permissivity of these bats to divergent HNVs and provide a framework for evaluating potential susceptibility across bat genera. Notably, *R*. *ferrumequinum* BKT1 and *R*. *leschenaultii* DemKT1 cells supported overall greater replication of rGhV_M-GS*_ than rNiV, suggesting a species-specific restriction of NiV. Previous studies have demonstrated that experimentally challenged *Rousettus aegyptiacus* bats do not support productive NiV or CedV infection *in vivo.*^60,61^ However, serological and metagenomic evidence suggests that *Roussettus* spp. bats in Africa harbor divergent HNVs,^62,63^ and a recent study identified two new HNV species in *R*. *leschenaultii* bats in China.^64^ For *Rhinolophus* spp., experimental infection with NiV has not been reported, leaving their compatibility with HNVs largely unexplored.^65^ Future studies will aim to identify mechanisms of post-entry restriction specific to NiV, but not GhV, in these cells.

Our *in vivo* work demonstrates that rGhV_M-GS*_ is attenuated in Syrian golden hamsters. EFNB3 receptor usage has been speculated to play a role in the neurotropism and neurovirulence associated with NiV and HeV infection.^17^ In support of this, recent *in vivo* work has demonstrated that Cedar virus, a non-pathogenic HNV which uses EFNB2 but not EFNB3, is unable to infect the central nervous system of experimentally infected IFNAR-knockout (KO) mice.^66^ However, CedV lacks V and W accessory proteins.^67^ In NiV, experimental KO of the V protein alone was sufficient to attenuate disease in hamsters and ferrets.^52,53^ As a result, it is difficult to disentangle the contributions of receptor usage and innate immune evasion that shape the restricted tropism and lack of pathogenicity in CedV.^67–70^

Because the rNiV_GhV-RBP_ chimera maintains authentic NiV replicative machinery and the full repertoire of innate immune antagonists, our findings support EFNB3 usage as a major determinant of HNV pathogenesis. More broadly, these results indicate that receptor usage and innate immune antagonism act in tandem to govern host tropism and immune evasion, with both cooperatively contributing to disease outcome. Furthermore, immunization with the rNiV_GhV-RBP_ chimeric virus elicited atypically broad antibody responses and protection against WT rNiV in hamsters, suggesting that presentation of heterotypic viral envelope proteins may be valuable for developing vaccine approaches to elicit polyclonal responses against divergent HNVs.

Our profiling of rGhV_M-GS*_ and rNiV susceptibility to small-molecule inhibitors aligns with previous studies demonstrating that the nucleoside analogs EIDD-2749, Remdesivir, and Favipiravir are active against NiV.^48,49,51^ Validating previous work in our rGhV TC-tr minigenome, the non-nucleoside allosteric inhibitor GHP-88309 was demonstrated to be effective against rGhV_M-GS*_ but not rNiV.^29^ The resistance of NiV to GHP-88309 reflects structural differences in the viral polymerase that prevent effective binding of the compound, consistent with prior studies.^49,71,72^ This study provides evidence of inhibition of HNV replication by the ribonucleoside analog EIDD-1931, the active form of molnupiravir. Future studies are warranted to validate the efficacy of molnupiravir *in vivo,* and to better determine if this drug, which has been employed in the clinic,^73^ may be appropriately repurposed to treat henipaviral infections.

Collectively, our work documents an underappreciated dimension of African bat henipavirus biology, providing experimental insights into host tropism, innate immune antagonism, and pathogenic potential of GhV. Comparative infection profiling of rNiV and rGhV_M-GS*_ across a variety of cell lines revealed that while rGhV_M-GS*_ exhibits limited replication with delayed kinetics in non-chiropteran cells, infection is more robust in bat-derived cells. Consistent with this attenuated phenotype outside of chiropteran hosts, experimental infection of Syrian golden hamsters with rGhV_M-GS*_ did not result in disease. Furthermore, we identify EFNB3 receptor usage as a major determinant of HNV pathogenicity by demonstrating that a chimeric, EFNB3-blind rNiV is completely attenuated *in vivo*. Together, these findings suggest that GhV may require further adaptation to achieve efficient replication in non-chiropteran hosts, and even in the event of spillover, its inability to utilize EFNB3 may limit disease severity. Nevertheless, the observation that primary human and porcine cells support even low-level rGhV_M-GS*_ replication underscores the importance of continued surveillance and experimental investigation to better anticipate and mitigate future spillover events involving African bat henipaviruses.

### Limitations of the study

Despite the insights gained from this study, we acknowledge certain limitations. The infectious clone of GhV M74a employed in this study likely diverges from authentic, circulating GhV in certain respects. Specifically, the HeV-derived Ldr28 sequence utilized in lieu of the unmapped genomic promoter may not reflect the authentic genomic promoter of GhV. With only a single reference sequence and a dearth of environmental isolates, we likewise cannot definitively say whether the non-canonical M-GS sequence in the GhV M74a reference genome is representative of circulating quasispecies or if it is an artifact of sequencing. Similar viral engineering was necessary to achieve the rescue of Lloviu virus from its reference sequence, which likewise lacked genomic ends and the necessary *cis*-acting elements due to sequencing artifacts.^74^ These difficulties highlight the challenges associated with recovering viruses from sequence alone. While rGhV_M-GS*_ did not cause disease in Syrian golden hamsters under the conditions tested, we cannot exclude the possibility that naturally circulating GhV variants may differ in regulatory elements that influence virulence or disease outcome. Thus, conclusions regarding GhV pathogenicity should be interpreted cautiously until additional genomic data or viral isolates become available.

## RESOURCE AVAILABILITY

### Lead contact

Requests for further information and resources should be directed to and will be fulfilled by the lead contact, Benhur Lee (benhur.lee@mssm.edu).

### Materials availability

All unique/stable reagents generated in this study are available from the lead contact.

### Data and code availability

- Numerical data underlying the figures in this manuscript are deposited in Mendeley Data: https://doi.org/10.17632/rjgcxt4cxr.1. Sequences of the rGhV reverse genetics plasmids have been deposited in GenBank under accession numbers GenBank: PX138233–PX138236. Whole-genome sequencing data of rGhV stocks are deposited in NCBI BioProject: PRJNA1284405. Long-read direct RNA sequencing data of GhV-infected cells are deposited in NCBI BioProject: PRJNA1307648.
- This paper does not report original code.
- Any additional information required to reanalyze the data reported in this paper is available from the lead contact upon request.

## STAR★METHODS

### EXPERIMENTAL MODEL AND STUDY PARTICIPANT DETAILS

### Animals

Syrian golden hamsters (*Mesocricetus auratus*) aged 5–6 weeks were obtained from Envigo (Indianapolis, IN, USA). Male and female hamsters were used, with sexes evenly distributed across experimental groups. The study was not powered to detect sex-specific differences in infection outcomes, and no sex-dependent effects were observed under the conditions tested. Hamsters were housed under standard laboratory conditions in an AAALAC-accredited BSL-4 facility with *ad libitum* access to food and water. All animal experiments were approved by the Institutional Animal Care and Use Committee (IACUC) at the University of Texas Medical Branch (protocol numbers 1302006 and 2204021) and were conducted in accordance with institutional and national guidelines for animal welfare. Animals were monitored daily for clinical signs of disease, and humane endpoints were applied as described in the method details.

### Cell culture and treatment

BSR-T7/5, Vero CCL81, HEK-293T, CHO-B2, and CHO-B3 cells were maintained under standard conditions as described in the method details. CHO-B2 and CHO-B3 cell line generation was previously described.^17,75^ Bat-derived cell lines were obtained from previously described sources^76–80^ and maintained at low passage. Primary human cells, including astrocytes, human umbilical vein endothelial cells, and human brain microvascular endothelial cells, were obtained from commercial vendors as specified. Primary human cells were commercially sourced and allocated to experimental conditions in parallel wells as described; no direct human participant enrollment was performed. Primary porcine kidney epithelial cells were obtained from commercial vendors as specified and maintained under conditions described in method details. Sex information for primary human cells, primary porcine cells, and bat-derived cell lines was not available and therefore could not be analyzed as a biological variable. BSR-T7/5 cells were routinely tested and confirmed negative for mycoplasma contamination using the MycoAlert Mycoplasma Detection Kit (Lonza Biosciences). Other cell lines were not routinely tested for mycoplasma contamination prior to experimentation. However, representative stocks of the bat-derived cell lines tested subsequently were confirmed negative for mycoplasma contamination. Cell line authentication was not independently performed; all cell lines were used at low passage and monitored routinely for morphology and growth characteristics.

### METHOD DETAILS

#### Maintenance of cell lines

BSR-T7/5 (CVCL_RW96), Vero CCL81 (CVCL_0059), HEK-293T (CVCL_0063), BKT1 (CVCL_YZ67), DemKT1 (CVCL_YZ68), ZFBK13–75, and EidNi/41.2 (CVCL_RX13) cells were maintained in Dulbecco’s modified Eagle medium (DMEM) supplemented with 10% fetal bovine serum (FBS, GeminiBio). ZFBS13–75A (CVCL_YZ77) and ZFBK11–97 (CVCL_YZ74) were maintained in RPMI 1640 media supplemented with 10% FBS. The generation of CHO pgsA-745 cells overexpressing EFNB2 or EFNB3 (CHO-B2 and CHO-B3) have been previously described,^17^ and were maintained in DMEM/F-12 media supplemented with 10% FBS. All bat cell lines were derived from kidney, with the exception of ZFBS13–75A, which is spleen-derived. Primary human cells were sourced from ScienCell Research laboratories, including human astrocytes (catalog #1800), human brain microvascular endothelial cells (catalog #1000), and human umbilical vein endothelial cells (catalog #8000). All primary cells were maintained at low passage, with HUVECs and HBMECs maintained in endothelial cell medium with kit (ScienCell catalog #1001) and HAs maintained in astrocyte medium with kit (catalog #1801). Normal human bronchial epithelial cells (Lonza, catalog #CC-2540) were grown in BEGM bronchial epithelial cell growth media with kit (Lonza, catalog #CC-3170). Small airway epithelial cells (Lonza, catalog #CC-2547) were maintained in SAGM small airway epithelial cell growth media with kit (Lonza, catalog #CC-3118). Primary porcine kidney epithelial cells (CellBiologics #P-6034) were maintained in complete epithelial cell medium with kit (CellBiologics #M6621). All cells were grown at 37°C in 5% CO_2_.

#### Design and cloning of reverse genetics plasmids

The full antigenomic sequence of rGhV strain Eid_hel/GH-M74a/GHA/2009 (NCBI Reference Sequence NC_025256.1) was fully synthesized (Bio Basic Inc.) and assembled into the pEMC vector. The non-coding region (NCR) between the N and P genes was duplicated to accommodate insertion of the GLuc-P2A-eGFP reporter, and there is an artificial NotI restriction site immediately downstream of the reporter gene’s stop codon. The HeV Ldr28 sequence (the terminal 3′-most 28 nucleotides of the HeV 3′ leader sequence) was inserted upstream of the reported GhV M74a sequence as previously described in minigenome.^29^ The viral antigenomic sequence is flanked on its 5′ terminal end by a sequence-specific hammerhead ribozyme element (5′- AGATCGGTCTGATGAGTCCGTGAGGACGAAACGGAGTCTAGACTCCGTC-3′) and on its 3′ terminal end by the HDV ribozyme, as has been detailed for our paramyxovirus reverse genetics systems.^44,81,82^ An optimal T7 promoter sequence lies upstream of the 5′ hammerhead ribozyme sequence, and a T7 terminator sequence lies downstream of the 3′ HDV ribozyme sequence. To restore the consensus motif in the M-GS sequence (M-GS*), a combination of restriction digest, PCR, and InFusion cloning were employed. Whole-plasmid sequencing confirmed that rGhV and rGhV_M-GS*_ antigenome plasmids (beyond reporter gene integration and modification of the M-GS) matched the GhV M74a reference sequence with the exception of a single-nucleotide change in the 3′ UTR of the GhV-N gene, in which there was a C1851T mutation.

The accessory plasmids encoding GhV-N, -P, and -L, and the rGhV TC-tr minigenome were generated as described previously.^29^ The native sequence of GhV-N, -P, and -L genes, respectively, were encoded into the pTM1 vector downstream of the EMCV IRES, between the SpeI and XhoI restriction sites, as previously described for NiV and HeV accessory plasmid construction.^83^ The ORF of the C protein was silenced in the GhV-P accessory plasmid by silently mutating its start codon. For some constructs, the N-GS in the rGhV TC-tr minigenome was replaced with the M-GS and M-GS* sequences, respectively. Sequences of the GhV reverse genetics plasmids have been deposited in NCBI GenBank (Accession numbers PX138233- PX138236).

The accessory plasmids for NiV (NiV-N, -P, and -L) were constructed and described previously.^44^ The rNiV (Malaysia strain UMMC1) encoding eGFP and *Gaussia* luciferase was rescued as previously described.^11,44,45^ The rNiV_GhV-RBP_ chimera was constructed by replacing the CDS of the NiV-RBP gene with the CDS of the GhV-RBP gene. This was achieved by digesting the WT rNiV antigenome plasmid with AfeI (upstream of NiV-RBP) and BsiWI (downstream of NiV-RBP) to remove the RBP gene. PCR was used to synthesize inserts between the AfeI site and the start codon of the RBP, the GhV-RBP CDS, and the stop codon of RBP through the BsiWI site; all inserts were designed with overlap for InFusion assembly. Cloning was achieved in all cases by a combination of restriction enzyme digest and InFusion-mediated fragment assembly (Takara Bio). All restriction enzymes were purchased from New England Biolabs, and all primers employed were synthesized by Millipore Sigma.

#### TC-tr minigenome assays

BSR-T7/5 cells were seeded one day prior to transfection in 24-well format, to achieve 70–80% confluence on the day of transfection. Twenty-four hours later, cells were co-transfected with plasmids encoding respective the rHNV TC-tr minigenome, codon-optimized T7 polymerase, and HNV-N, -P, and -L as previously described.^29^ All transfections used Lipofectamine LTX and PLUS according to the manufacturer recommendations. 1.39 micrograms of respective TC-tr minigenome, 0.40 micrograms of codon-optimized T7 polymerase, 0.49 micrograms of HNV-N, 0.32 micrograms of HNV-P, 0.16 micrograms of HNV-L, 1.27 mL of PLUS, and 2.04 mL of LTX per reaction constituted each minigenome reaction. For all transfections, mastermixes were prepared which contained the TC-tr minigenome, HNV-N, HNV-P, and codon-optimized T7; these master mixes were then aliquoted into equal parts before adding plasmid encoding HNV-L or plasmid encoding GFP. Plasmid encoding GFP was used in lieu of HNV-L as a control to determine background signal nonspecific to the viral replicase. At 24 HPT, media was replaced on plates. Quantification of mCherry events and nanoluciferase assays were conducted at 48–72 h post-transfection, depending on the experiment.

To measure nanoluciferase activity, TC-tr minigenome cells were lysed and processed using the Nano-Glo HiBiT Lytic Detection System (Promega Corporation), according to the manufacturer recommendations. Cell lysate was transferred to white 96-well plates and RLUs were measured on a Cytation 3 plate reader (BioTek) using a gain of 125 and an integration time of 1.0s. Raw RLUs were then normalized to the average RLUs of the no-L (GFP) control to determine the vRdRp-dependent signal above background. A Celigo Imaging Cytometer (Nexcelom) was used to image the entire plate of TC-tr minigenome cells in the red channel. The total number of mCherry positive events were quantified using Celigo software analysis of the red channel.

#### Rescue and amplification of full-length rGhV

350,000 BSR-T7/5 cells per well were seeded into 6-well plates. Twenty-four hours later, when cells were approximately 70% confluent, the media on each plate was exchanged for 1.5mL of fresh DMEM +10% FBS containing 1x Nucleic Acid Transfection Enhancer (Invivogen). Transfection reactions were prepared by diluting 4.0 micrograms of plasmid encoding codon-optimized T7 polymerase, 5.0 micrograms of pTM1_T7-GhV-N, 3.2 micrograms of pTM1_T7-GhV-P, and 1.6 micrograms of pTM1_T7-GhV-L in 200 mL of OptiMEM. 21 mL of Mirus TransIT-LT1 were then added to the transfection reaction and gently mixed. After a 30 min incubation at room temperature, transfection complexes were added dropwise to BSR-T7/5 cells. At 24 h post-transfection (HPT), media was exchanged on every well with 1.5mL of fresh DMEM +10% FBS containing 1x Nucleic Acid Transfection Enhancer (Invivogen). The cells were then moved into high containment (Galveston National Laboratory BSL-4, University of Texas Medical Branch) for transfection of the antigenome. The rGhV antigenomic plasmid was diluted in 200 mL of OptiMEM before adding 21 mL of Mirus TransIT-LT1 to the reaction. After gently mixing, transfection complexes were incubated at room temperature for 30 min prior to being added dropwise to the replicase-transfected cells.

Rescue cells were monitored daily for signs of CPE and reporter gene expression. To maintain cell viability, media was exchanged every other day (DMEM containing 10% FBS and 1x Nucleic Acid Transfection Enhancer). Upon observation of obvious secondary spread, supernatant was harvested daily with replacement and frozen at −80°C. Stocks of rGhV_M-GS*_ were made by infecting Vero CCL81 and CHO-B2 cells, respectively, with P0 supernatant. Upon observation of widespread (>90%) virus amplification in flask, as determined by CPE and GFP expression, infected flasks were freeze-thawed once and supernatant was harvested, clarified by brief centrifugation, and frozen at −80°C.

#### Rescue and amplification of rNiV and rNiV_GhV-RBP_

Rescue and amplification of rNiV (Malaysia strain) constructs was conducted as previously described.^11,44,45^ BSR-T7/5 cells were seeded at a density of 400,000 cells per well into six-well plates. A day later, when cells had reached 80% confluence, transfection reactions were prepared by diluting 7.0 micrograms of rNiV or rNiV_GhV-RBP_ antigenomic plasmid, 2.0 micrograms of codon optimized T7 polymerase, 2.5 micrograms of pTM1_T7-NiV-N, 1.6 micrograms of pTM1_T7-NiV-P, and 0.8 micrograms of pTM1_T7-NiV-L in 200 mL of OptiMEM. Cells and plasmid mixes were then moved to the high containment, where 21 mL of Mirus TransIT-LT1 was added to the transfection reaction and gently mixed. After a 30 min incubation at room temperature, transfection complexes were added dropwise to the BSR-T7/5 cells. Rescue cells were monitored daily for signs of infection (CPE and reporter gene signal). Upon observation of obvious secondary spread, media was harvested with replacement and stored at −80°C or used to infect Vero CCL81 cells. Upon widespread virus amplification in the flask, as determined by CPE and GFP expression, the flask was freeze-thawed once and supernatant was harvested, clarified by brief centrifugation, and frozen at −80°C.

#### Virus work and biosafety

All work with full-length, infectious rNiV and rGhV was conducted in class II BSC at the Galveston National Laboratory BSL-4, and at The Robert E. Shope, MD, Laboratory BSL-4 at the University of Texas Medical Branch (UTMB). All samples requiring removal from high containment were rendered inactivated/non-infectious using institutionally-approved protocols, and samples were rigorously validated to be non-infectious prior to removal.

#### Virus titration

Stocks and infectious samples of rNiV and rNiV_GhV-RBP_ were titrated by plaque assay. 250,000 cells/well of Vero CCL81 cells were seeded in 12-well format. Twenty-four hours later, virus was serially diluted in DMEM +10% FBS and cells were infected in low volume (100 mL) for 1 h at 37°C. Following this 1 h incubation, semi-solid overlay was added onto each well (1x MEM +0.5% methylcellulose +2%FBS) and plates were incubated at 37°C until assay endpoint. Plates were fixed in crystal violet buffered in 10% formalin and read at 72 HPI for rNiV or 5 DPI for rNiV_GhV-RBP_.

Because rGhV_M-GS*_ did not generate sufficient CPE for plaque assay, we titrated virus on highly permissive CHO-B2 cells via 50% tissue culture infectious dose (TCID_50_). 96-well plates were seeded with 15,000 cells/well of CHO-B2 cells. The next day, virus was serially-diluted in DMEM/F-12 + 10% FBS and incubated on CHO-B2 cells overnight at 37°C. At 24 HPI, media was replaced on all wells to minimize residual *Gaussia* luciferase signal from the inoculum. At 72HPI, supernatant was harvested for *Gaussia* luciferase assay. Infected wells were determined by RLU signal and titer (TCID_50_/mL) was determined using the Reed-Muench method.^84^ All TCID_50_ assays were conducted using 4 to 6 technical replicates per sample.

#### Virus growth kinetics

To determine the growth kinetics of rGhV_M-GS*_ and rNiV, 200,000 cells/well of CHO-B2 cells were seeded into 12-well format. The following day, ells were infected in triplicate, using low-volume inoculum (100 mL), with either rGhV_M-GS*_ or rNiV at an MOI of 0.05 for 1 h at 37°C. Plates were gently rocked every 15 min to prevent drying and to ensure even distribution of the inoculum. The titer of rGhV_M-GS*_ was converted to PFU/mL by multiplying the TCID_50_/mL titer by 0.7. After 1 h incubation, viral inoculum was removed, and the cells were gently washed with 1mL of DPBS to remove residual GLuc and unbound virus. The DPBS was removed, and 1.5 mL of DMEM:F12 + 10% FBS was added to the wells. We then harvested and froze 0.5 mL of supernatant at −80°C, which served as the “0 HPI” sample. After this, 0.5 mL of supernatant was harvested every 24 h with replacement and frozen at −80°C. All timecourse samples were subsequently thawed for use in virus titration and *Gaussia* luciferase assays.

#### Interferon stimulated response reporter gene assays

The NiV and GhV-P, -V, and -W genes were subcloned from the pTM1_T7-NiV-P and pTM1-T7-GhV-P plasmids, with PCR used to append a triple FLAG tag onto the N terminus of each gene (CloneAmp, TakaraBio). InFusion Cloning (TakaraBio) was used to assemble the respective gene products into pCAGGs expression vector.

For luciferase assays, HEK-293T cells were transfected with: (1) the construct encoding the respective P, V, W, or empty vector (EV) sequence, (2) a plasmid containing Firefly luciferase gene downstream of an IFN-stimulated response element (pISRE-TA-Luc), and (3) a construct encoding the Renilla luciferase protein (pRL-Luc). Transfections were performed using lipofectamine LTX and PLUS (Invitrogen).

To test antagonism of MDA5-mediated signaling, plasmid encoding FLAG-tagged MDA5 under the CAGG promoter was co-transfected into cells in parallel with the plasmids above. At 24 HPT, cells were lysed in 1x passive lysis buffer (Promega). To test antagonism of IFN-β mediated signaling, at 24 HPT, cells were treated with 1000 units of human IFN-β and incubated at 37°C for 6 h. After the 6 h incubation period, cells were lysed in 1x passive lysis buffer. Lysates were employed in SDS-PAGE and Dual-Glo Luciferase Assay System (Promega). Firefly luciferase values were normalized to the Renilla control to account for differences in transfection.

#### Western blotting

Protein content in lysates were quantified using Bradford Assay, and equal amounts of protein were combined with 1x Laemelli buffer with DTT and boiled for 5 min 95°C before being transferred onto ice. Samples were loaded onto pre-cast, 4–20% mini-PROTEAN polyacrylamide gels, and proteins were resolved via electrophoresis in the Mini-PROTEAN Tetra cell (BioRad). Proteins were transferred to PVDF membranes using the Trans-Blot Turbo transfer system (BioRad). Membranes were blocked with phosphate-buffered saline blocking buffer (LI-COR; 927–700001) and then probed with the indicated antibodies. Antibodies against FLAG (Sigma-Aldrich, F3165), GAPDH (Cell signaling, 2118), STAT1 (Cell signaling, 14994), STAT2 (Cell signaling, 72604), HA (GeneTex, GTX115044), and COXIV (Cell signaling, 4850) were employed. Secondary antibodies conjugated to Alexa Fluor 647 or Alexa Fluor 546 were used to detect respective primary antibodies. For western blotting of samples from the GhV TC-tr minigenome transfections, proteins were transferred to nitrocellulose membranes and probed for HiBiT using the Nano-Glo HiBiT blotting system (Promega) according to manufacturer instructions. All blots were imaged using a ChemiDoc MP imaging system (Bio-Rad).

#### Co-immunoprecipitation assays

HEK-293T cells were transfected with 200ng of respective FLAG-tagged viral protein or EV using Lipofectamine LTX and PLUS. At 24 HPT, cells were treated with 1000 U/mL IFN-β for 6 h, lysed, and lysates were subjected to immunoprecipitation using anti-FLAG beads (Sigma-Aldrich, A2220). Western blots were performed as described for other assays, with IP eluates and whole-cell lysates probed to detect FLAG (viral proteins), STAT1, and STAT2.

#### Timecourse for primary cells

To determine the growth kinetics of rGhV_M-GS*_ and rNiV in primary human cells, 100,000 cells/well of HAs, HUVECs, or HBMECs were seeded into poly-L-lysine coated 24-well format. The following day, cells were infected in triplicate, using low-volume inoculum (100 mL), with either rGhV_M-GS*_ or rNiV at an MOI of 0.1 for 1 h at 37°C. Plates were gently rocked every 15 min to prevent drying and to ensure even distribution of the inoculum. The titer of rGhV_M-GS*_ was converted to PFU/mL by multiplying the TCID_50_/mL titer by 0.7. After the 1 h incubation, viral inoculum was removed and cells were gently washed with 0.5 mL of DPBS to remove residual GLuc and unbound virus. The DPBS was removed, and 0.6 mL of respective cell-specific media was added to the wells. We then harvested and froze 0.1 mL of supernatant at −80°C, which served as the “0 HPI” sample. After this, 20 mL of supernatant was harvested every 24 h with replacement and was frozen at −80°C. All timecourse samples were subsequently thawed for use in virus titration (0HPI vs. endpoint) and *Gaussia* luciferase assays.

For timecourse studies with NHBE and SAEC, 25,000 cells/well were seeded into 96-well format. For PPKE, 15,000 cells/well were seeded into 96-well format. Cells were then infected with rNiV or rGhV_M-GS*_ at an MOI of 0.1 for 1 h at 37°C. Cells were then rinsed with 0.1 mL of DPBS, and 120 mL of respective media was added to each well. 20 mL of media was then harvested and frozen at −80°C, serving as the “0 HPI” timepoint. After this, 20 mL of supernatant was harvested every 24 h with replacement and was frozen at −80°C. All timecourse samples were subsequently thawed for use in *Gaussia* luciferase assays.

#### Timecourse for immortalized cells

For timecourse studies using immortalized cells (CHO-B2, CHO-B3, ZFBS13–75A, ZFBK13–75, ZFBK11–97, FBKT1, BKT1, and DemKT1) 15,000 cells/well were seeded into 96-well format. A day later, cells were then infected with rNiV or rGhVM-GS* at an MOI of 0.05, as titered on CHO-B2 cells, for 1 h at 37°C. After the 1 h infection, cells were rinsed with 0.1 mL of DPBS (to remove residual *Gaussia* luciferase from the virus stock), and 120 mL of respective media was added to each well. 20 mL of media were then harvested and frozen at −80°C, serving as the “0 HPI” timepoint. After this, 20 mL of supernatant was harvested with replacement at denoted timepoints and frozen at −80°C. All timecourse samples were subsequently thawed at the same time for use in *Gaussia* luciferase assays.

#### *Gaussia* luciferase assays

*Gaussia* luciferase activity in the supernatant reflects the amount of secreted luciferase produced from the viral GLuc-P2A-eGFP reporter cassette, providing a quantitative surrogate for viral gene expression and replication. Relative light units (RLUs) correspond to the luminescence generated by *Gaussia* luciferase in the sample and increase proportionally with viral replication. *Gaussia* luciferase assays were carried out using the Pierce Gaussia Luciferase Glow Assay kit (Thermo Fisher Scientific), according to manufacturer instructions. In general, 20 mL of sample were gently mixed with substrate diluted in *Gaussia* glow assay buffer in opaque, black-bottom 96-well plates. After a ten minute incubation at room temperature, plates were read for luminescence on a Cytation5 (Biotek) plate reader.

For timecourse studies of rNiV and rGhV, 20 mL of supernatant were collected with replacement at each time point and frozen at −80°C. Once all timepoints had been collected, samples were thawed simultaneously and used for *Gaussia* luciferase assay as described above. The RLUs at each timepoint were then normalized to the average RLUs of each condition at 0 HPI to gauge fold-change in RLUs over time.

#### *In vivo* hamster challenges

All animal studies were approved by the IACUC committee at UTMB (protocol # 1302006, and 2204021). Groups of five 5–6 week old, equal-sex Syrian golden hamsters (Envigo, Indianapolis, IN, USA) were challenged as described using respective virus diluted in volume of 100 mL via the intraperitoneal (IP) or intranasal (IN) route. Back titration of inocula was performed on the challenge day when possible and is reported. Hamsters were monitored at least once daily until clinical symptoms onset, and up to 3 times a day during peak disease. Body weights were taken daily for the first 9–10 days, then every 3 days for the remainder of the study. Moribund animals (determined via clinical scoring or displaying greater than 20% weight loss) were euthanized. Clinical scoring included change in breathing (labored, irregular) and a bloody nose, while criteria of a neurological involvement included aggressive behavior, any form of paralysis, ataxia, head tilt, and/or seizure. Retroorbital bleeds were carried out at 2, 4, 6, and 8 DPI. At time of euthanasia, terminal serum was first collected via cardiac puncture. For each manipulation (viral infection or biosampling), animals were anesthetized with isoflurane (Piramal, Bethlehem, PA).

#### Detection and quantification of neutralizing antibody titers from serum

To determine the titer of neutralizing antibodies from animal sera against rNiV or rNiV_GhV-RBP_, plaque reduction neutralization tests (PRNTs) were performed in 12-well format. Serum was heat inactivated at 56°C for 30 minutes prior to serial dilution in MEM with 2%FBS. Diluted sera was incubated with a dose of 40 PFU of rNiV or rNiV_GhV-RBP_ for one hour at 37°C. Vero cells were then infected with the virus:serum mix, and a standard plaque assay was conducted. PRNT_50_ was defined as the antibody dilution yielding 50% reduction in plaques.

To determine the titer of neutralizing antibodies from animal sera against rGhV_M-GS*_, luciferase reduction neutralization tests (LRNTs) were performed in 96-well format. Serum was heat inactivated at 56°C for 30 minutes prior to serial dilution in DMEM/F-12 with 2% FBS. Diluted sera was incubated with a dose of 1500 PFU of rGhV_M-GS*_ for one hour at 37°C. CHO-B2 cells were then infected with the virus:serum mix. Media was changed at 24 HPI, and supernatant was collected at 72 HPI for *Gaussia* luciferase assay. LRNT_50_ was defined as the antibody dilution yielding a 50% reduction in RLUs relative to mock.

#### Drug inhibition assays

For drug inhibition assays, 15,000 cells/well of CHO-B2 or primary human astrocyte cells were seeded in 96-well format and infected the next day with rNiV or rGhV_M-GS*_ at an MOI of 0.05. Serially-diluted compounds were added at the time of infection. At 24 HPI, the supernatant was replaced with fresh DMEM +10% FBS containing respective compound dilutions. At 48 HPI, 20 mL of supernatant was collected and transferred to an opaque, black-bottom plate for *Gaussia* luciferase assay. Raw RLUs were normalized to the DMSO control and multiplied by 100 to determine signal in each condition relative to vehicle. All drug inhibition assays were conducted in at least biological triplicate, and IC_50_ values were calculated in GraphPad Prism software by nonlinear regression of the [inhibitor] vs. normalized response. Compounds employed in this study include EIDD-2749 (MedChemExpress, HY-146246), GHP-88309 (Sigma Aldrich, SML2997), EIDD-1931 (MedChemExpress, HY-125033), Remdesivir (MedChemExpress HY-104077), and Favipiravir (Cellagen Technology, C8705–5). All compounds were diluted according to manufacturer recommendations.

#### Long read direct RNA sequencing

CHO-B2 cells were infected with rGhV_M-GS*_ at an MOI of 0.05. At 72 HPI, total RNA was collected using 0.5 mL of TRIzol reagent. Direct-zol RNA Miniprep kits were used to extract total RNA (Zymo Research). RNA was eluted in ultra-pure water and stored at −80°C. RNA samples were demonstrated to be non-infectious in CHO-B2 cells, which showed no reporter gene activity nor CPE for two passages in high containment. Total RNA was prepared for sequencing library using the Direct RNA sequencing kit (Oxford Nanopore Technologies, SQK-004), and libraries were sequenced on an RNA sequencing flow cell (FLO-MIN004RA). Basecalling was achieved using Dorado 7.6.7 in the MinKNOW 24.11.8 software, and reads were aligned to the rGhV_M-GS*_ genome using Minimap2.^85^ P editing was quantified using mPileup in Samtools, selecting for reads with inserts of Gs only.^86^ Raw sequencing reads have been deposited in NCBI BioProject ID PRJNA1307648.

#### Sequencing of GhV stocks

Whole-genome sequencing of the four rGhV stocks was conducted using a metagenomic next-generation sequencing approach as previously described.^87^ Libraries were sequenced on an Illumina NextSeq 2000 platform with 2 × 150bp reads. Raw reads were trimmed and quality filtered with fastp (v0.23.4).^88^ Filtered reads were then used for variant calling using the RAVA workflow (default parameters) and the plasmid map sequence as a reference (https://github.com/greninger-lab/RAVA_Pipeline/tree/2025-06-30_GhV_Griffin_et_al).^89,90^ Raw sequencing reads have been deposited in NCBI BioProject: PRJNA1284405.

#### RT-qPCR for EFNB2 and EFNB3

Total RNA was extracted from respective bat cell lines using TRIzol reagent and the Direct-zol RNA Miniprep kit (Zymo Research). RNA was eluted in ultra-pure water and stored at −80°C. The Primer-free LunaScript RT Master Mix Kit (New England Biolabs) was used for first-strand cDNA synthesis using gene-specific primers targeting EFNB2, EFNB3, or GAPDH. A no-RT control was employed in parallel. Following cDNA synthesis, equal volumes of cDNA were used for qPCR using the Luna Universal qPCR Master Mix kit (New England Biolabs). For qPCR, gene specific primers were utilized targeting either a 189-basepair region of the EFNB2 gene (FWD: 5′-AGGGACTCCGTGTGGAAGTA-3’; REV: 5′-AGAGTCCACTTTGGGGCAAAT-3′), a 169-basepair region of the EFNB3 gene (FWD: 5′- TCTCCGCTTCACCATCAAGT-3’; REV: 5′-TCGGAGAAGCACCTTCATGC-3′), or a 166-basepair region of the GAPDH gene (FWD: 5′-TCAAGGGCATCCTGGGCTA-3’; REV: 5′- ACCACCCTGTTGCTGTAGCCAA-3′). Signal was captured on a C1000 Touch Thermal Cycler (Bio-Rad), and data were exported for analysis. All qPCR reactions were conducted in at least biological duplicate. Relative gene expression was determined by first calculating the ΔCT of EFNB2 (CT_EFNB2_ - CT_GAPDH_) and the ΔCT of EFNB3 (CT_EFNB3_ - CT_GAPDH_). Then, all ΔCT values for EFNB2 or EFNB3, respectively, were normalized to ZFBK13–75 by calculating ΔΔCT(ΔCT_X_ – AVG[ΔCT_ZFBK13–75_]). Values were plotted as 2^−ΔΔCT^.

#### Microscopy

For microscopic imaging, the nuclei of cells were stained using NucBlue Live ReadyProbes Reagent (ThermoFisher) at a concentration of 2 drops/mL; cells were incubated in NucBlue-containing solution for at least 30 min prior to imaging. Microscopic images were collected using an Olympus IX83 microscope. All images at a given timepoint were collected using the same settings for each channel. Images were subsequently processed in ImageJ software.

#### *In silico* analyses of GhV gene CDS

The CDS of all GhV M74a genes were evaluated through a combination of *in silico* approaches. The amino acid sequences of each GhV protein were aligned to their homologues from NiV, strain UMMC1 (GenBank: AY029767.1), HeV strain HeV/Australia/1994/Horse18 (GenBank: MN062017.1), Cedar virus strain CG1a (GenBank: JQ001776.1), and Angavokely virus (GenBank: ON613535.1). Alignments were conducted in MegaX using Clustal Omega, and were manually inspected. Amino acid percent identity tables were generated using Clustal Omega webserver (https://www.ebi.ac.uk/jdispatcher/msa/clustalo). Phylogenies were created in MegaX using the neighbor-joining method with 1000 bootstrap iterations.^91^ Structural comparisons between GhV and NiV were conducted by either: (1) comparing resolved structures available on PDB, or (2) comparing predicted models generated using AlphaFold3. All structural analyses were conducted in UCSF ChimeraX, and RMSD was determined using the matchmaker function.

#### Phylogeny of bat cytochrome *b* gene

The evolutionary history of chiropteran cytochrome *b* was inferred by using the Maximum Likelihood method and JTT matrix-based model.^92^ The tree with the highest log likelihood (−1568.10) is shown. The percentage of trees in which the associated taxa clustered together is shown next to the branches. Initial tree(s) for the heuristic search were obtained automatically by applying Neighbor-Join and BioNJ algorithms to a matrix of pairwise distances estimated using the JTT model, and then selecting the topology with superior log likelihood value. The tree is drawn to scale, with branch lengths measured in the number of substitutions per site. This analysis involved 5 amino acid sequences. Sequences of Cytochrome *b* were obtained from uniprot for each species: *Rhinolophus ferrumequinum* (accession O21298), *Eidolon helvum* (accession A0A7G3WDD6), *Rousettus leschenaultii* (accession B9VHX7), *Pteropus dasymallus* (accession Q9G6M3), and *Epomophorus gambianus* (accession G1C2G6). There were a total of 379 positions in the final dataset. Evolutionary analyses were conducted in MEGA X.^91^

#### Production of pseudotyped HNVpp-Rluc

HEK293T cells (4 × 10^6^) were seeded in collagen-coated 10-cm^2^ dishes and transfected at 24hr post-seeding using BioT (Bioland Scientific) with 10 micrograms each of codon-optimized plasmids encoding homotypic HNV-F and RBP. At 16 HPT, cells were infected with VSV-ΔG-Rluc at a multiplicity of infection (MOI) of 10. Following two washes in PBS, fresh DMEM (10% FBS) supplemented with 1:10,000 anti-VSV-G monoclonal antibody (EB0010, Kerafast) was added. Supernatants were collected and clarified (1200 rpm, 5 min) at 48 HPI. GhVpp were concentrated by ultracentrifugation over a 20% sucrose cushion (pelleted using 25,000 rpm for 2hr at 4°C) and resuspended in PBS. Infections were performed at a titer yielding at least a 3-log Rluc signal over Rluc background.

#### HNVpp neutralization assay

Neutralization assays were conducted using titrated HNVpp-Rluc incubated with serial dilutions of polyclonal sera for 1 h at 37°C. Polyclonal sera were heat-inactivated at 56°C for 30 min prior to use. The mixtures were added to U-87 MG cells (3 × 10^4^ cells/well). At 24 h post-infection, cells were lysed (Promega, E2810), and Renilla luciferase activity was measured using a Cytation 3 (BioTek).

#### Flow cytometry-based surface binding assay

HEK293T cells (4 × 10^6^) were seeded in 6-well plates during 24h and transfected (BioT, Bioland Scientific) with 2 μg/well codon-optimized plasmids encoding FLAG-tagged HNV-F, or RBP-HA. At 24 h post-transfection, cells were harvested mechanically in PBS and optionally stained with Violet viability dye (1:10,000, ThermoFisher, L34955) for 20 min in the dark. Cells were washed in PBS, pelleted down and stained with primary antibodies diluted in FACS buffer (1x PBS, 15% FBS, 17 mM EDTA) for 1 h at 37°C. Following two washes (FACS buffer), cells were incubated with Alexa 647-conjugated secondary antibodies (1:2000) for 1 h at room temperature in the dark. Washed twice, and resuspended in PBS. HNV glycoproteins were stained independently to measure expression using 1:1000 AU1 (Sigma Aldrich, F3165) or HA (Novusbio, NB600–363) mAb with corresponding secondary antibody Alexa 647. Flow cytometry was performed on an Attune NxT, using a no-primary control for gating. Data were analyzed in FlowJo v10.9.0, and GMFI values were plotted in GraphPad Prism v9.0.0. Binding was quantified as geometric mean fluorescence intensity (GMFI) after background subtraction.

### QUANTIFICATION AND STATISTICAL ANALYSIS

All statistical analyses were performed using GraphPad Prism software. Details regarding statistical tests, number of replicates, what n represents, measures of center and dispersion, and significance thresholds are detailed in the corresponding figure legends. Animals were randomly assigned to experimental groups. No blinding was performed. No data or animals were excluded from analysis unless otherwise stated.

## Supplementary Material

1

SUPPLEMENTAL INFORMATION

Supplemental information can be found online at https://doi.org/10.1016/j.celrep.2026.117074.

## Figures and Tables

**KEY RESOURCES TABLE:** 

REAGENT or RESOURCE	SOURCE	IDENTIFIER
Antibodies		
ANTI-FLAG^®^ M2 antibody, Mouse monoclonal	Sigma-Aldrich	Cat# F3165; RRID:AB_259529
GAPDH (14C10) Rabbit Monoclonal Antibody	Cell Signaling Technology	Cat# 2118; RRID:AB_561053
Stat1 (D1K9Y) Rabbit Monoclonal Antibody	Cell Signaling Technology	Cat# 14994; RRID:AB_2737027
Stat2 (D9J7L) Rabbit Monoclonal Antibody	Cell Signaling Technology	Cat# 72604; RRID:AB_2799824
HA tag Rabbit Polyclonal Antibody	GeneTex	Cat# GTX115044; RRID:AB_10622369
COX IV (3E11) Rabbit Monoclonal Antibody	Cell Signaling Technology	Cat# 4850; RRID:AB_2085424
Bacterial and virus strains		
Recombinant Nipah Virus (rNiV) GLuc-P2A-eGFP reporter	This study	Constructed in-house based on Malaysia strain UMMC1 (GenBank: AY029767.1)
Recombinant Ghana virus (rGhV) GLuc-P2A-eGFP reporter	This study	Constructed in-house based on strain Eid_hel/GH-M74a/GHA/2009 (NCBI Reference Sequence: NC_025256.1)
Recombinant Ghana virus encoding M-GS* (rGhV_M-GS_*) GLuc-P2A-eGFP reporter	This study	Constructed in-house based on strain Eid_hel/GH-M74a/GHA/2009 (NCBI Reference Sequence: NC_025256.1). Plasmid sequence available (GenBank: PX138236).
Recombinant Nipah Virus encoding GhV-RBP (rNiV_GhV-RBP_) GLuc-P2A-eGFP reporter	This study	Constructed in-house based on Malaysia strain UMMC1 (GenBank: AY029767.1) and GhV strain Eid_hel/GH-M74a/GHA/2009 (NCBI Reference Sequence: NC_025256.1)
Biological samples		
Primary human astrocytes (HAs)	ScienCell Research laboratories	Cat# 1800
Primary human umbilical vein endothelial cells (HUVECs)	ScienCell Research laboratories	Cat# 8000
Primary human brain microvascular endothelial cells (HBMECs)	ScienCell Research laboratories	Cat# 1000
Primary human small airway epithelial cells (SAECs)	Lonza Bioscience	Cat# CC-2547
Primary normal human bronchial epithelial cells (NHBECs)	Lonza Bioscience	Cat# CC-2540
Primary porcine kidney epithelial cells (PPKECs)	CellBiologics	Cat# P-6034
Chemicals, peptides, and recombinant proteins		
EIDD-2749 (4′-Fluorouridine)	MedChemExpress	Cat# HY-146246
GHP-88309	Sigma Aldrich	Cat# SML2997
EIDD-1931 (β-D-N4-hydroxycytidine; NHC)	MedChemExpress	Cat# HY-125033
Remdesivir (GS-5734)	MedChemExpress	Cat# HY-104077
Favipiravir (T-705)	Cellagen Technology	Cat# C8705-5
Recombinant Human IFN-beta Protein	R&D Systems	Cat# 8499-IF
Critical commercial assays		
Pierce^™^ Gaussia Luciferase Glow Assay Kit	Thermo Scientific	Cat# 16161
Dual-Glo^®^ Luciferase Assay System	Promega	Cat# E2940
Nano-Glo^®^ HiBiT Lytic Detection System	Promega	Cat# N3040
Direct-zol^™^ RNA Miniprep Kit	Zymo Research	Cat# R2052
Direct RNA Sequencing Kit	Oxford Nanopore Technologies	Cat# SQK-RNA004
Deposited data		
Whole-genome sequencing of rGhV_M-GS_* stocks	This study	NCBI BioProject: PRJNA1284405
Direct RNA sequencing (Nanopore) of rGhV_M-GS_* infected CHO-B2 cells	This study	NCBI BioProject: PRJNA1307648
Numerical data underlying figures	This study	Mendeley Data: https://doi.org/10.17632/rjgcxt4cxr.1
Experimental models: cell lines		
BSR-T7/5	Karl-Klaus Conzelmann	RRID:CVCL_RW96
Vero CCL-81	ATCC	Cat# CCL-81; RRID:CVCL_0059
HEK-293T	ATCC	Cat# CRL-3216; RRID:CVCL_0063
BKT1	Maeda et al., 2008	RRID:CVCL_YZ67
FBKT1	Maeda et al., 2008	RRID: CVCL_YZ69
ZFBK11-97	Maruyama et al., 2014	RRID: CVCL_YZ74
ZFBS13-75A	Maruyama et al., 2016	RRID: CVCL_YZ77
EidNi/41.2	Susanne et al., 2011	RRID:CVCL_RX13
ZFBK13-75	van Tol et al., 2023	N/A
CHO-B2	Negrete et al., 2006	N/A
CHO-B3	Negrete et al., 2006	N/A
Experimental models: organisms/strains		
Syrian golden hamster (*Mesocricetus auratus*)	Envigo	N/A
Oligonucleotides		
EFNB2 Primer for RT-qPCR (Forward)	MilliporeSigma	5′-AGGGACTCCGTGTGGAAGTA-3′
EFNB2 Primer for RT-qPCR (Reverse)	MilliporeSigma	5′-AGAGTCCACTTTGGGGCAAAT-3′
EFNB3 Primer for RT-qPCR (Forward)	MilliporeSigma	5′-TCTCCGCTTCACCATCAAGT-3′
EFNB3 Primer for RT-qPCR (Reverse)	MilliporeSigma	5′-TCGGAGAAGCACCTTCATGC-3′
GAPDH Primer for RT-qPCR (Forward)	MilliporeSigma	5′-TCAAGGGCATCCTGGGCTA-3′
GAPDH Primer for RT-qPCR (Reverse)	MilliporeSigma	5′-ACCACCCTGTTGCTGTAGCCAA-3′
Recombinant DNA		
Recombinant Ghana virus reverse genetics plasmids (N, P, L, and antigenome constructs)	This study	GenBank: PX138233-PX138236
Codon-optimized T7 RNA polyermase	Yun et al., 2015	Addgene #65974
Software and algorithms		
GraphPad Prism 10.3.1	GraphPad Software	https://www.graphpad.com/
UCSF ChimeraX	UCSF	https://www.cgl.ucsf.edu/chimerax/
ImageJ	ImageJ	https://imagej.net/ij/
MEGA X	MEGA	https://www.megasoftware.net/
AlphaFold3 Server	Google DeepMind and Isomorphic Labs	https://alphafoldserver.com/
MinKNOW 24.11.8	Oxford Nanopore Technologies	https://nanoporetech.com/document/experiment-companion-minknow
Dorado (basecalling)	Oxford Nanopore Technologies	Release 7.6.7
Minimap2	Li et al., 2018	https://github.com/lh3/minimap2
SAMtools	Danecek er al., 2021	https://www.htslib.org/
FlowJo v10.9.0	BD Biosciences	https://www.flowjo.com/
RAVA: Reference-based Analysis of Viral Alleles	Lin et al., 2019	https://github.com/greninger-lab/RAVA_Pipeline/tree/2025-06-30_GhV_Griffin_et_al
Celigo Software v5.5.0.0	Nexcelom Bioscience	Celigo.com

## Highlights

- Reverse genetics enables the *de novo* recovery of Ghana virus
- Ghana virus is Ephrin-B3 blind and causes no disease in Syrian golden hamsters
- Ghana virus replicates efficiently in bat cells but is attenuated in human cells
- Loss of Ephrin-B3 receptor usage attenuates Nipah virus pathogenicity
